# Complete Primate Skeleton from the Middle Eocene of Messel in Germany: Morphology and Paleobiology

**DOI:** 10.1371/journal.pone.0005723

**Published:** 2009-05-19

**Authors:** Jens L. Franzen, Philip D. Gingerich, Jörg Habersetzer, Jørn H. Hurum, Wighart von Koenigswald, B. Holly Smith

**Affiliations:** 1 Forschungsinstitut Senckenberg, Frankfurt, Germany; 2 Naturhistorisches Museum Basel, Basel, Switzerland; 3 Museum of Paleontology and Department of Geological Sciences, University of Michigan, Ann Arbor, Michigan, United States of America; 4 Natural History Museum, University of Oslo, Oslo, Norway; 5 Steinmann-Institut für Geologie, Mineralogie und Paläontologie, Universität Bonn, Bonn, Germany; 6 Museum of Anthropology, University of Michigan, Ann Arbor, Michigan, United States of America; University of Wisconsin, United States of America

## Abstract

**Background:**

The best European locality for complete Eocene mammal skeletons is Grube Messel, near Darmstadt, Germany. Although the site was surrounded by a para-tropical rain forest in the Eocene, primates are remarkably rare there, and only eight fragmentary specimens were known until now. Messel has now yielded a full primate skeleton. The specimen has an unusual history: it was privately collected and sold in two parts, with only the lesser part previously known. The second part, which has just come to light, shows the skeleton to be the most complete primate known in the fossil record.

**Methodology/Principal Findings:**

We describe the morphology and investigate the paleobiology of the skeleton. The specimen is described as *Darwinius masillae* n.gen. n.sp. belonging to the Cercamoniinae. Because the skeleton is lightly crushed and bones cannot be handled individually, imaging studies are of particular importance. Skull radiography shows a host of teeth developing within the juvenile face. Investigation of growth and proportion suggest that the individual was a weaned and independent-feeding female that died in her first year of life, and might have attained a body weight of 650–900 g had she lived to adulthood. She was an agile, nail-bearing, generalized arboreal quadruped living above the floor of the Messel rain forest.

**Conclusions/Significance:**

*Darwinius masillae* represents the most complete fossil primate ever found, including both skeleton, soft body outline and contents of the digestive tract. Study of all these features allows a fairly complete reconstruction of life history, locomotion, and diet. Any future study of Eocene-Oligocene primates should benefit from information preserved in the *Darwinius* holotype. Of particular importance to phylogenetic studies, the absence of a toilet claw and a toothcomb demonstrates that *Darwinius masillae* is not simply a fossil lemur, but part of a larger group of primates, Adapoidea, representative of the early haplorhine diversification.

## Introduction

**Figure 1:**
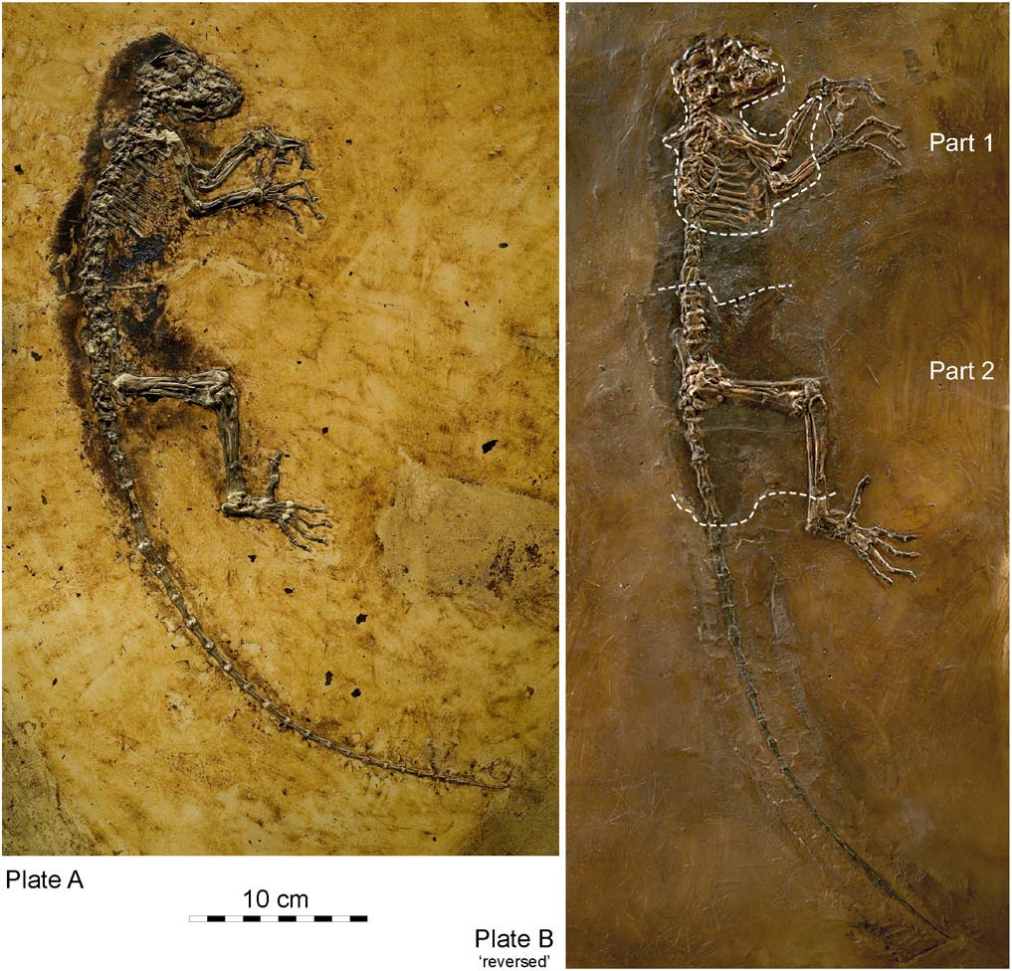
*Darwinius masillae*, new genus and species, from Messel in Germany. (A)— Plate A (PMO 214.214) showing holotype skeleton in right lateral view. (B)— Plate B (WDC-MG-210) left side of holotype (reversed for comparison with plate A). Plates show part and counterpart of the same skeleton. Plates have different museum numbers because they are in different museum collections. Note the exceptional completeness of the articulated skeleton in plate A, with left and right hands and the right foot complete, including distal phalanges, and the tail complete to the tip. Stained matrix shows the soft-tissue body outline. Abdomen contains organic remains of food in the digestive tract. All of plate A and parts 1 and 2 on plate B (enclosed in dashed lines) are genuine; remainder of plate B was fabricated during preparation.

**Figure 2 pone-0005723-g002:**
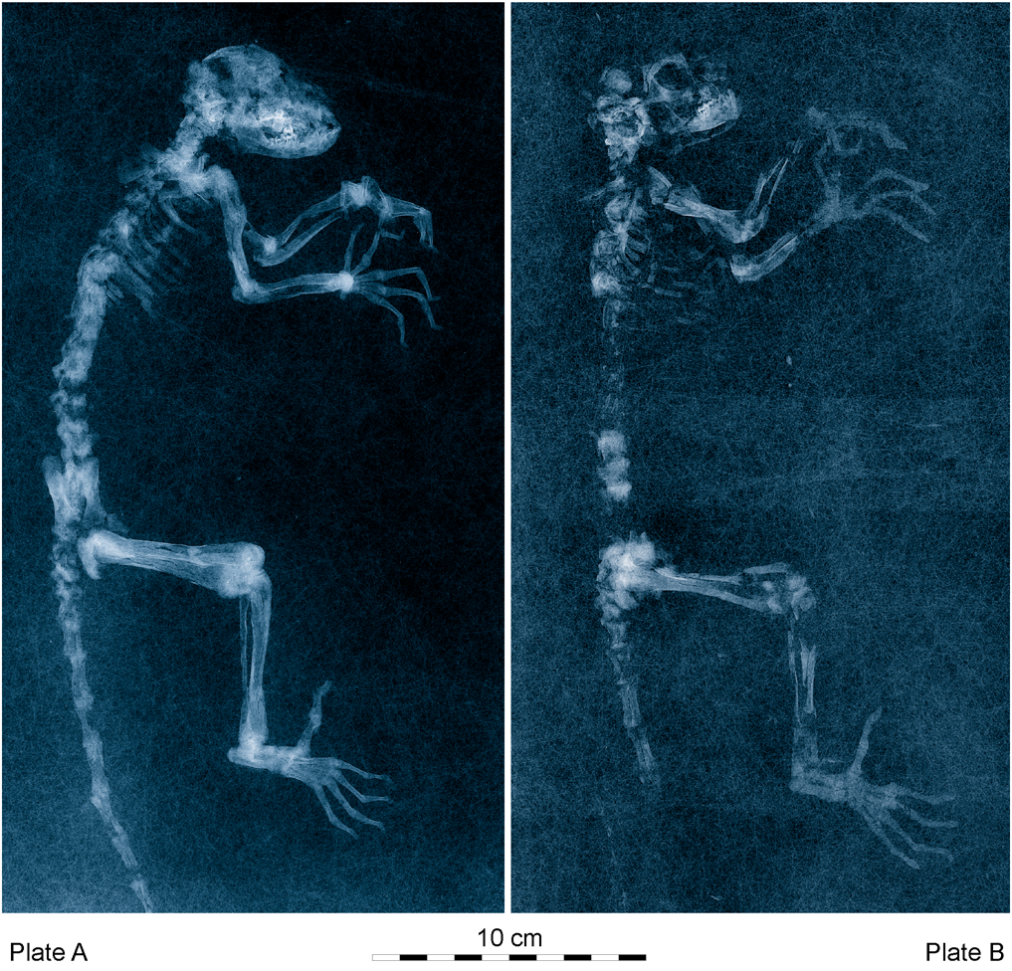
Radiographs of the type specimen of *Darwinius masillae*, new genus and species, from Messel in Germany. Relative positions and museum numbers as in Figure 1. Radiographs show that all of plate A is genuine, while cranium, thorax, upper arms (part 1), and lumbus, pelvis, base of tail, and upper legs (part 2) of plate B are genuine.

A set of extraordinary circumstances produced one of the most complete skeletons of a fossil primate ever recovered, here described as a new genus and species *Darwinius masillae*. The holotype is a juvenile that died at the margin of a volcanic lake in a paratropical rain forest and was preserved in Middle Eocene sediments of Messel, Germany (Grube Messel or ‘Messel pit,’ herein simply Messel). The fossil was apparently unearthed in 1983 by private collectors who split and eventually sold two parts of the skeleton on separate plates: the lesser part (herein plate B) was restored and in the process partly fabricated to make it look more complete. This was eventually purchased for a private museum in Wyoming, and then described by one of us who recognized the fabrication [1]. The more complete part (plate A; Figs. 1–2) has just come to light, and it now belongs to the Natural History Museum of the University of Oslo (Norway). When made available for study, plate A was immediately recognizable as the complete complementary and unaltered counterpart of plate B.

The new specimen, like some other Messel finds, is complete even to distal phalanges and terminal tail vertebrae. Moreover, it was exceptionally preserved during fossilization, retaining soft tissue outlines and contents of the digestive tract. Like other Messel fossils, however, the skeleton is lightly crushed and must be examined in place. Individual bones and teeth cannot be physically removed to examine individually, a difficulty we have partially overcome with innovative CT imagery.

The specimen is a juvenile, but erupting teeth indicate the developmental age and enable prediction of further growth of the body and limbs. The completeness of the fossil allows us to reconstruct aspects of life history, diet, and locomotion that are difficult to study in fossils. In addition, the skeleton enables identification of characteristics routinely used to distinguish strepsirrhine and haplorhine primates. Our focus here is on morphology and paleobiology, but the skeleton has interest for primate phylogeny as well. The skeleton's features clarify morphologies that have been given critical weight in primate phylogeny, and call into question accepted wisdom about the origin of higher primates.

### Eocene primates

The first primates of modern aspect appeared at the beginning of the Eocene epoch, about 55 m.y. before present. Two superfamilies can be recognized from the beginning: (1) Tarsioidea, including Eocene Omomyidae and Microchoeridae and living *Tarsius*; and (2) Adapoidea, including Eocene Notharctidae and Adapidae, with later representatives but no living primates. Tarsioidea are generally smaller, with estimated body weights less than 500 g; Adapoidea are generally larger, with estimated body weights greater than 500 g [2]–[4]. Within Notharctidae, the subfamily Cercamoniinae (sometimes considered a family Cercamoniidae) has special interest because of its shortened, robust dentaries, reduced antemolar dentition, and interlocking canines with monkey-like honing premolars [5], all features that may foreshadow anthropoids. Cercamoniinae include primates as widely dispersed as *Protoadapis* and *Cercamonius* from France, *Europolemur* from Germany, *Caenopithecus* from Switzerland, *Mahgarita* from Texas, and *Aframonius* from Egypt.

### Messel

Messel is a maar lake deposit. The basin in which the deposit accumulated formed during a volcanic explosion. It filled with water, which seemingly, one way or another, accumulated gases that poisoned animals individually, episodically, or periodically [6]–[8]. The result is a diverse fauna of exceptionally preserved insects, fishes, amphibians, reptiles, birds, and mammals [9]–[12].

The Messel locality is inferred to represent a paratropical Eocene rain forest. Primates are rare faunal elements at Messel, in spite of the rainforest habitat, and only eight primate specimens are known, all previous finds fragments of partial skeletons (Table 1) [13]–[19], [1]. In all, three primate species are known from Messel: *Europolemur koenigswaldi, Europolemur kelleri*, and a species formerly identified as *Godinotia neglecta* (see below). All belong to Notharctidae and subfamily Cercamoniinae. No tarsioid primates have been found at Messel, but they are common in contemporary deposits elsewhere in Europe and should be present. The Messel fauna belongs to the early middle Eocene or earliest Geiseltalian, MP11 [20] with a calculated radiometric age of ca. 47 Ma based on a basalt fragment coming from an underlying volcanic chimney [21].

**Table 1:** Systematic synopsis of the primate remains from Messel, with museum collection numbers, Messel primate number, Messel grid coordinates (for map see Fig. S1).

Present designation	Preserved Parts	Collection	Number and locality	Description
*Darwinius masillae* (holotype)	Complete skeleton, same individual as No. 6b - **plate A**	Natural History Museum Oslo PMO 214.214 (found by private collectors in 1982)	**No. 6 A** [?7H, 7I, -8H]	This study
*Darwinius masillae* (same individual as holotype)	Two fragments of a skeleton, same individual as No. 6a - **plate B**	WDC-MG -210 (found by private collectors in 1982)	No. **6 B** [?7H, 7I, -8H]	[1], [24]
*Europolemur koenigswaldi*(holotype)	Partial skeleton, comprising skull, thorax, vertebral column, os sacrum, and the right anterior limb	SMF-ME 1228 (found in 1982)	No. **2** [12M–13M]	[10], [15], [16]
*Europolemur koenigswaldi*	fragmentary mandible with cheek-teeth	SMF-ME 2986 (found in 1997)	No. **7** [9E]	[1], [83]
*Europolemur koenigswaldi*	Fragment of the pelvis, complete hindlimbs of both sides, and a complete tail vertebral column.	SMNK-Me1125 (found in 1990)	No. **5** [9D]	[38]
*Europolemur kelleri* (holotype)	Complete, but dorso-ventrally compressed skull	SMF-ME-3379 (found by private collectors)	No. **8** [?]	[19]
*Europolemur kelleri*	Pelvis, baculum, and both hindlimbs	HLMD-Me 7430 (found in 1975)	No. **1** [7G]	[13]
*Europolemur kelleri*	Right arm, forearm and hand	SMF-ME 1683 (found in 1987)	No. **4** [10L]	[17], [84], [85], [86]
*Europolemur* sp.	hindlimbs of both sides and a baculum	SMNK III 1641 (found in 1984)	**No. 3** [11E]	[14]

### History of the specimen

In order to comprehend how part and counterpart of the same individual fossil can have such different histories, it is essential to understand how fossils at Messel are collected and preserved. Here complete fossil mammal skeletons are well preserved, along with those of fish, amphibians, reptiles and birds. These almost always lie on bedding planes of the laminated sediment. During the early years of excavation for fossils, between 1971 and 1985, mining for oil shale had extensively exposed sediments. Once mining was finished, plans arose to use the open pit as a garbage dump. With this in mind, early excavations for fossils were necessarily rushed, and less attention was paid to careful bed by bed collecting of fossils. Large blocks of the oil shale were removed and split along bedding planes using long knives. The presence of fossils enhances the splitting.

Before starting preparation of a plate for study, the surface damaged by splitting must be embedded in epoxy or polyester resin. Then the as yet unexposed lateral surface of the plate is prepared to expose the lateral side of the little-damaged fossil. This procedure is necessary as dehydration of the oil shale destroys a fossil. The ideal situation is when part and counterpart are mirror images, and both right and left sides of the animal can be prepared equally well. Alternatively, the split can be such that most bones remain on one plate, leaving their natural cast on the counterpart plate.

From what we know of the present fossil, it was privately collected at Messel in 1983, at the foot of what is known as the Schildkrötenhügel (Turtle Hill) see Fig. S1, although the exact horizon is unknown (personal communication from previous owner of plate A, Thomas Perner, Bad Homburg).

Plate B (Figs. 1,2), originally described by Franzen [18] as the sixth Messel primate (Table 1), had a curious history. It was purchased in 1991 by Dr. Burghard Pohl for the Wyoming Dinosaur Center at Thermopolis, Wyoming. This plate holds a partial skeleton viewed from the left side, embedded in a plate of polyester. Franzen [18] showed that some of the specimen is real, while substantial parts were faked to give an illusion of greater completeness. Working from what was available, Franzen attributed the specimen to the species “*Pronycticebus neglectus*” (Thalmann, Haubold & Martin, 1989) described from Geiseltal [22]. He first placed the species in *Caenopithecus*, and then assigned it to a new genus *Godinotia* [1].

Plate A (Figs. 1,2) described here, became available for sale and was purchased in 2007 by the Natural History Museum of the University of Oslo (Norway). This plate, showing a skeleton from the right side, proves to be the hitherto unknown and much more complete counterpart of the Wyoming Plate B. Careful study and comparison of the new and more complete plate indicates that the specimen cannot belong to *Godinotia neglectus* (see below).

The Oslo specimen, plate A, clarifies exactly which parts of plate B were faked, including notably, hands and feet (where some proportions of constructions may have been based on reversed photos of A) and the tail vertebral column. Traces on the surrounding polyester resin background suggest that a cast of the tail of another mammal was inserted into plate B. Additional parts such as the vertebrae between sections 1 and 2 as well the nasal part of the skull on plate B were simply fabricated

The almost complete skeleton on plate A has been well prepared, and it also lies on a polyester resin background. Preservation is unique. The cranium is compressed, but a combination of plates A and B shows virtually the entire dentition. Plate A also shows almost the entire right side of the body and several parts of the left side of the body that are missing on plate B. Only the distal part of the left leg is missing on both plates. Thus the skeleton of *Darwinius masillae* is much more complete than any known for *Notharctus* Gregory 1920 [23], and in addition it is unique in exhibiting the entire soft body outline as well as contents of the digestive system [24].

## Methods

**Figure 3:**
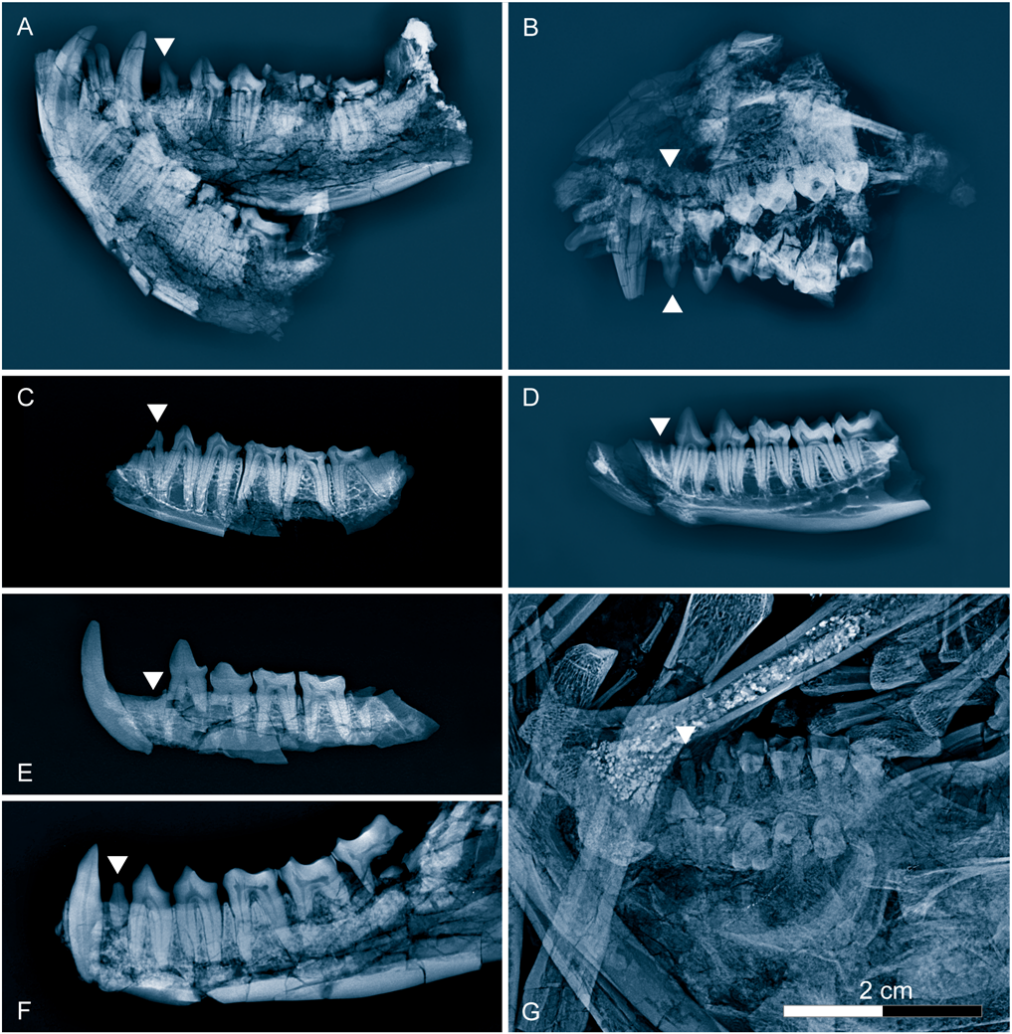
Radiographic comparison of middle Eocene primates from Geiseltal in eastern Germany. (A)— *Europolemur klatti* (Weigelt, 1933), GMH CeIV-3656, left and right mandible with I_1–2_, C_1_, P_2_-M_3_d and I_1–2_, C_1_, P_3_-M_3_s. (B)— *Europolemur klatti* (Weigelt, 1933), GMH LeoI-4233, part of the skull with upper dentition, which is part of the holotype. (C)— *Europolemur klatti* (Weigelt, 1933), GMH XXXVII-120, fragmentary left mandible with double-rooted P_2_, P_3–4_, and heavily worn M_1–3_. (D)— *Europolemur klatti* (Weigelt, 1933), GMH XXII-1, right mandibular ramus with P_3_-M_3_ and alveoli for a double rooted P_2_. (E)— *Protoadapis ignoratus* (Thalmann, 1994), GMH XXII-549, part of type specimen, fragment of right mandible with C_1_, P_3–4_, M_1_, alveoli of P_2_ and M_3_ (mirrored). (F)— *Protoadapis weigelti* Gingerich, 1977, GMH XXII-624, right mandibular ramus with P_3_-M_3_, root of a small single-rooted P_2_ and alveolus of C_1_, which is isolated (mirrored). (G)— *Godinotia neglecta* (Thalmann, Haubold & Martin, 1989), holotype (GMH L-2), detail: palate containing M^3^-P^3^s and d, and the small unicuspid and one-rooted P^2^s. Arrows show the position of P^2^/P_2_. Geiseltal primates come from Middle Eocene zones MP12 and 13, slightly later in time than those from Messel (MP11). *Godinotia neglecta*, like *Darwinius masillae*, is distinguished from *Europolemur klatti* by the presence of small, straight, single-rooted P^2^.

Study of the compressed skeleton was facilitated by X-radiography and microcomputerized tomography (CT):

1. Contact microradiographs were made with conventional X-ray sources (Faxitron 43804 X-ray cabinet, and Faxitron 43856A X-ray cabinet, Hewlett Packard, USA) on a 25 micron storage screen (SR-HD-IP, Fuji, Japan), combined with a laser scanning digitizer (HD-CR 35 NDT, Duerr-NDT, Germany).
2. Microradiographs were enlarged by direct projection of the specimen using a microfocus X-ray tube with 10-micron resolution (FXT 100.52, Feinfocus/Yxlon, Germany) on a real-time digital sensor (C7942 CK12, version modified for small bones, Hamamatsu, Japan). Moderately enlarged microradiographs (1.9×) of comparative primate specimens (Fig. 3) were made with a conventional clinical digital mammography system (Mammomat Novation with enlargement set, Siemens, Germany).
3. CT images were obtained using an industrial Micro CT System (RayScan 200 XE, RayScan Technologies, Germany). The microfocus X-ray tube makes it possible, in principle, to achieve resolutions below 10 microns when small probes a few cm in size are used. However, the principal *Darwinius* plate is large (plate A), and it cannot be separated into smaller parts for CT analysis. For the whole plate, the maximum resolution was 430 microns, even using micro CT. This problem was overcome using a special algorithm (‘region of interest’ micro CT) on the RayScan apparatus, which increased the resolution to 68 microns. Artefacts of this algorithm are progressive fusion and loss of contrast of bones and teeth in the images, due to averaging of originally different densities. However, compromises can be found that are still acceptable at this resolution. Image processing of CT-data was undertaken with VGStudio MAX 2.0.1 (Volume Graphics, Germany).

**Figure 4:**
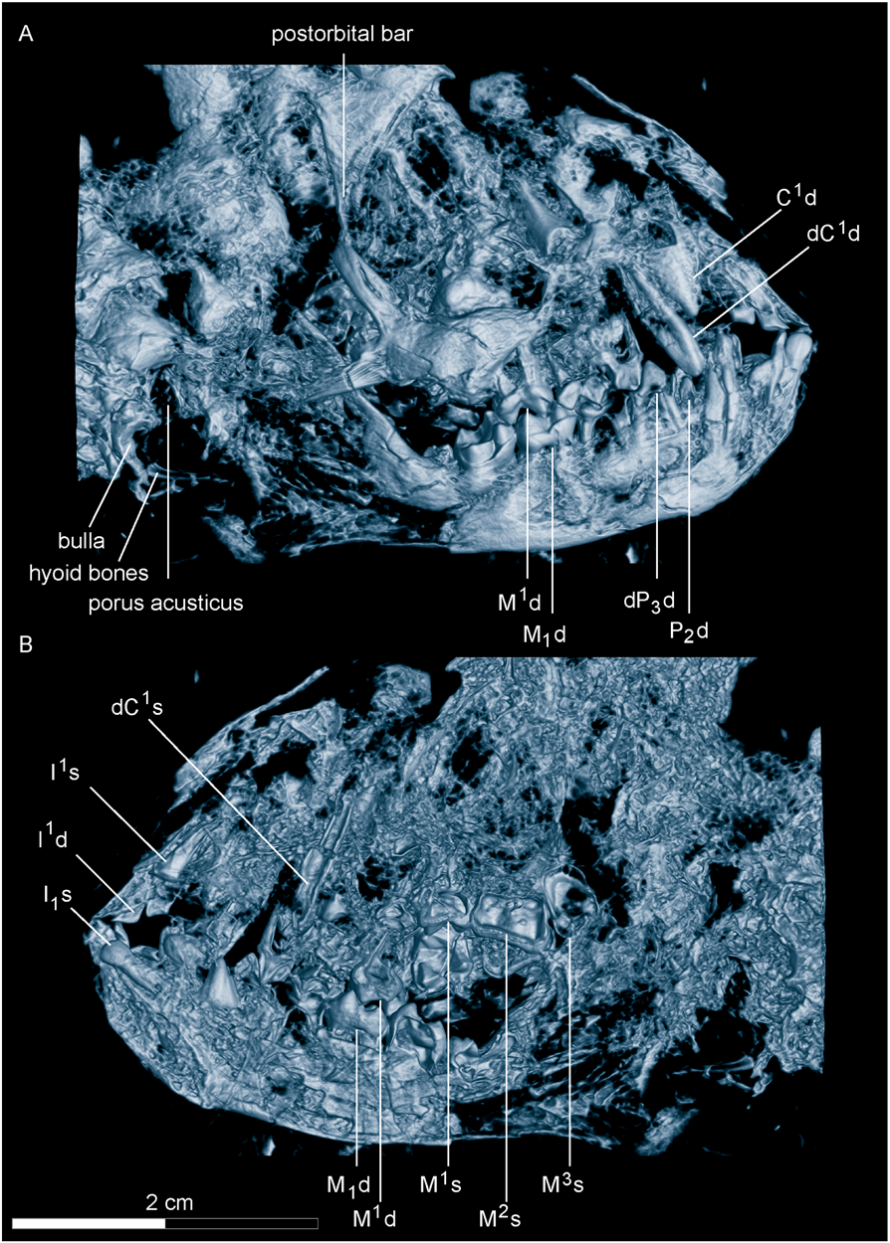
Micro-CT of the skull of *Darwinius masillae*, new genus and species. (A)— CT image of the skull in plate A, viewed from the right side. (B)— CT image of the skull in plate A, viewed from the left side. Note the presence of a postorbital bar, parts of the auditory bulla below the acoustic opening, and possible hyoid bones. Tooth homologies are mapped in greater detail in Figure 6 and sutures in S2.

Mapping of developing teeth was done using ArcGIS. First a high-resolution digital photograph of the dentition visible on the surface of plate A was mapped, tooth by tooth, using good light and a binocular microscope. The high-resolution digital X-ray was geo-referenced using landmarks visible in the photograph and X-ray. This permitted identification of some teeth that were not visible on the surface. Next in sequence a shaded CT image of the same region (Fig. 4A), a reversed shaded CT image of the same region viewed from the back side of plate A (Fig. 4B), a reversed photograph of the surface of plate B [1: fig. 4] and a reversed X-ray image of plate B were geo-referenced. Each tooth could be viewed, mapped, and checked by toggling between these superimposed images. In this way virtually all teeth and developing teeth in both plates and from all jaw quadrants were identified unambiguously.

Measurements of the holotype of *Darwinius masillae* n.gen. n.sp. were made using calipers, with the aid of a binocular microscope or hand lens. Comparisons with other specimens from Messel were made in the Senckenberg Museum, Frankfurt am Main, while comparisons with specimens from Geiseltal were made at the Geiseltalmuseum in Halle.

William Jungers (Stony Brook, New York) provided an extensive set of comparative measurements for multivariate analysis of skeletal proportions. Comparisons with the postcranial skeletons of modern primates were made using skeletons in the Senckenberg Museum (Frankfurt): *Eulemur mongoz* (SMF-M34725), *Varecia variegata* (SMF-M38471), *Avahi laniger* (SMF-M34718), *Loris* sp. (SMF-M10780), *Callithrix jacchus* (SMF-M59340, and -343), and *Cercopithecus neglectus* (SMF-M59230) and the University of Michigan Museum of Zoology (Ann Arbor): *Saguinus oedipus* (UMMZ 156437), *Saguinus mystax* (UMMZ 160148), *Callicebus moloch* (UMMZ 125576), *Cebus capucinus* (UMMZ 77296), and *Cebus apella* (UMMZ 126129). *Tarsius* sp., *Callithrix* sp and *Saimiri sciureus* skeletons were measured on University of Michigan Museum of Paleontology specimens (UMMP 139 and unnumbered).

Comparisons with *Notharctus osborni* refer to specimens described by Gregory [23] and a cast housed in the Department of Messel Research at the Forschungsinstitut Senckenberg at Frankfurt am Main.

### Terminology

Identification of teeth follows conventional nomenclature, with capital letters I, C, P, and M, representing incisors, canines, premolars, and molars. Superscripts indicate upper teeth. Subscripts indicate lower teeth. Deciduous teeth are prefaced with a lowercase d. When distinguished, left and right skeletal elements and teeth are followed by an *s*, for sinister or left, or a *d*, for dexter or right. The anatomical nomenclature follow [25].

Institutional abbreviations: HLMD-Me: Hessisches Landesmuseum Darmstadt, Messel-Collection; PMO: Geological Museum, Natural History Museum, University of Oslo, Norway. SMF-ME: Senckenberg Museum Frankfurt, Messel Collection; SMNK-Me: Staatliches Museum für Naturkunde Karlsruhe, Messel Collection; UMMP: University of Michigan Museum of Paleontology vertebrate collection; UMMZ: University of Michigan Museum of Zoology mammal collection; WDC-MG: Wyoming Dinosaur Center, Messel Grube collection.

## Results

Systematic Paleontology

Order Primates Linnaeus, 1758

Suborder Euprimates Hoffstetter, 1977

Family Notharctidae Trouessart, 1879

Subfamily Cercamoniinae Gingerich, 1975

***Darwinius*** **new genus**

### 

#### Type species

*Darwinius masillae* n.gen., n.sp.

#### Derivatio nominis

Honoring Charles Darwin on the occasion of his 200th birthday.

***Darwinius masillae*** **new species**

#### Holotype

By monotypy plate A, (PMO 214.214) with counterpart (plate B WDC-MG-210).

#### Derivatio nominis

*Masilla* = Messel in the Codex of the Lorsch monastery, 800 AD.

#### Type locality

Messel, near Darmstadt (South Hessen, Germany); geographic coordinates are: 49°55′7″ North, 8°45′22″ East.

#### Type horizon and age

Messel Formation (middle part of section), early Middle Eocene or early Geiseltalian (MP 11), ca. 47 Ma [20]–[21].

#### Diagnosis

M^1^and M^2^ display a well developed hypocone but no mesostyle. A metaconule is lacking. The M_1_ and M_2_ show a small trigonid and a very broad talonid. In the permanent dentition, P^1^/P_1_ have been lost whereas P^2^/P_2_ are unicuspid and uniradical, especially reduced in the maxilla. The lower segments of the anterior and posterior limbs are conspicuously short and robust. The phalanges are elongated. A toilet or grooming claw is not present. Molars of *Darwinius masillae* are distinct in morphology and intermediate in size between those of contemporary species of *Periconodon* and *Europolemur*.

#### Differential diagnoses

*Darwinius masillae* differs from species of *Europolemur* Weigelt, 1933 (Geiseltal-obere Mittelkohle and Messel) in having a very small, single-rooted P^2^/P_2_, whereas P^1^/P_1_ are completely reduced (lost).

Differs from *Caenopithecus lemuroides* Rütimeyer, 1862 (Egerkingen γ [26]) in being smaller and having upper molars that lack a mesostyle (postcranial skeleton of *Caenopithecus* is unknown except for an isolated talus; see below).

Differs from *Cercamonius brachyrhynchus* (Stehlin, 1912), from Prajous (Quercy Phosphorite deposits) in having a mandibular ramus that is mesially not as deep, a trigonid of M_1_ that is mesiodistally longer, and a talonid of M_1–2_ that is larger and broader. M_1_ and M_2_ have a separate metastylid cusp not seen in *Cercamonius*.

Differs from *Godinotia* Franzen, 2000b (Geiseltal-untere Mittelkohle) in having relatively shorter and more robust limbs.

#### Discussion

When Franzen described the counterpart specimen (plate B) and assigned it to *Godinotia neglecta* from Geiseltal [1], the permanent dentition of the Messel specimen was only represented by a fragmentary left M_1_ and an incomplete forelimb and lower leg without hands and feet. He therefore did not recognize the difference of limb proportions, basing his determination mainly on the similar degree of reduction of the antemolar dentition. In 1994 similarities of the dentition led him to assign Geiseltal and Messel specimens to the genus *Caenopithecus* described by Rütimeyer in1862 from Egerkingen γ [18], [27]. Now that the completely preserved right side of the Messel specimen (plate A) is known and described herein, it is clear that *Darwinius masillae* n.gen., n.sp. differs considerably from the type specimen of *Godinotia neglecta* in the postcranial skeleton and in particular, the limb proportions. Moreover, its dentition is clearly different from that of *Europolemur koenigswaldi* as well as *E. kelleri* from Messel and it differs from that of *Caenopithecus lemuroides* from Egerkingen in lacking a mesostyle on the upper molars.

The limb proportions of *Europolemur kelleri* Franzen, 2000a, *E. koenigswaldi* Franzen, 1988, and the North American *Notharctus osborni* Gregory, 1920, are similar, whereas the limbs of *E. klatti* Weigelt, 1933, from Geiseltal are unknown. The dentition of *E. kelleri*, *E. koenigswaldi* and *E. klatti* (type species) correspond so well, that there is no doubt that they belong to the same genus. Radiographs demonstrate that all species of *Europolemur* match each other in possessing unicuspid but two-rooted P^2^/P_2_, while that of *Godinotia neglecta* and that of *Darwinius masillae* are small, straight and one-rooted, almost remnants in the maxilla (Fig. 3). The type specimen of *G. neglecta* from Geiseltal clearly differs from *E. kelleri*, *E. koenigswaldi*, and *D. masillae* in having very gracile limb bones (Figs. 1–3). The postcranial skeleton of *E. klatti* is little known save for an isolated astragalus, calcaneum, and atlas, the species determination of which is uncertain [22: 50, 62–65, fig. 2.20].

### Description

#### Cranium

(Figs. 4, S2). The cranium in plate A is seen from the right side, while that in plate B is seen from the left [1: 290–293]). Bones and teeth are well preserved in both, but plate A is more complete. The profile of the face shows that the rostrum was relatively short, the face steep and the orbit large (see below). Measurements are listed in Table 2, and Appendix S1.

**Table 2:** Measurements of the skull and postcranial skeleton of the holotype of *Darwinius masillae*, n. gen., n. sp.

Skeletal element	Measurement (mm)	Remarks
**Cranium**		
Cranial length	**52.0**	Total skull length
Orbital diameters	**11.5**×**16.5**	Width and height
M^1^ crown	**3.80**×**4.65**	Length and width; measured on CT reconstruction
M_1_ crown	**4.05**×**2.90**×**3.30**	Length, trigonid width, talonid width; measured on CT reconstruction
M_2_ crown	**3.90**×**3.20**×**3.75**	Length, trigonid width, talonid width; measured on CT reconstruction
**Postcranium**		
Thorax	**61.0**	Sum of thoracic centrum lengths as articulated
Lumbus	**60.0**	Sum of lumbar centrum lengths as articulated
Scapula	**24.7**	Maximum length
Humerus	**46.7**	Maximum length
Radius	**36.5**	Maximum length
Hand	**46.0**	Measured from base of wrist to most distal phalanx
Ilium	**33.0**	Length measured from center of acetabulum
Femur	**65.5**	Maximum length
Tibia	**65.2**	Maximum length
Foot	**67.0**	Length measured from end of calcaneum to most distal phalanx
**Skeleton as a whole**		
Vertebral column	**ca. 53 cm**	Proximal atlas to end of tail
Total length with skull	**ca. 58 cm**	Skull plus vertebral column
Head and body length	**ca. 24 cm**	Without tail

Based on Micro-CT reconstructions of teeth and x-radiographs of the skeleton.

#### Rostrum and orbit

Nasale: The anterior parts of the nasals are not preserved. The ventral suture with the premaxilla is about one-third of the length of the suture with the maxilla and lachrymal (or median process of maxilla, see below). Following the impressions, the right nasal extends mesially to above I^2^ whereas the left ends above the border between I^1^ and I^2^. The right nasal contains three similar sized slit-like nasal foramina. The most caudal one is situated above the anterior rim of the orbit. The most mesial one occurs above the tip of the deciduous upper canine.

**Figure 5:**
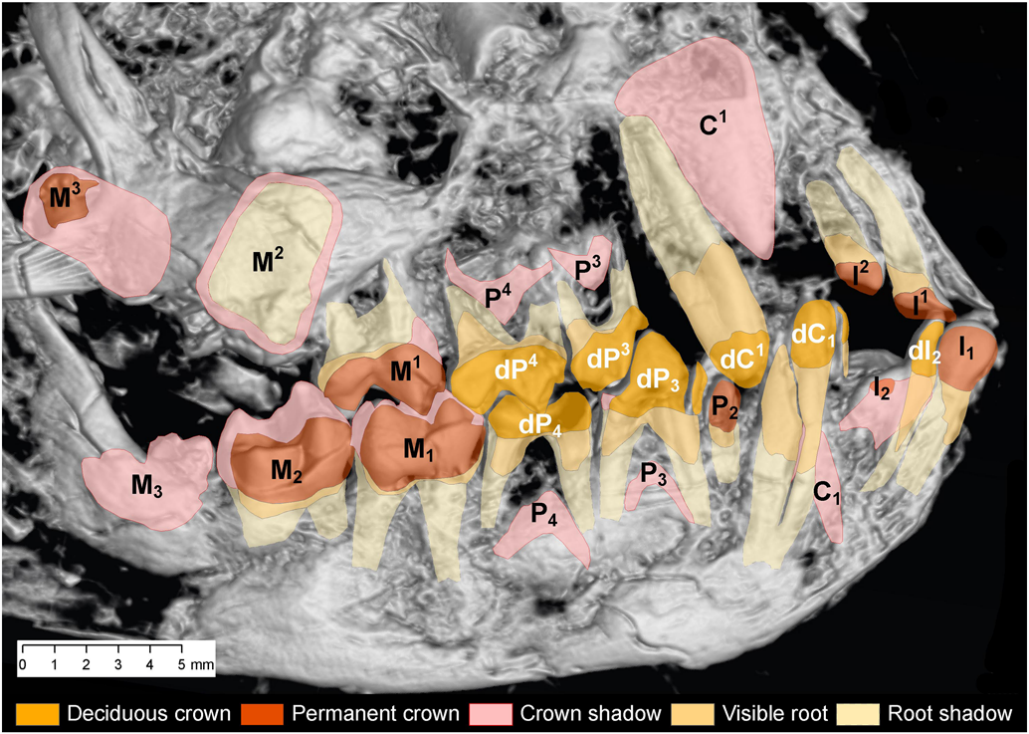
Map of deciduous and permanent teeth of the right side in the skull of *Darwinius masillae*, new genus and species. Deciduous dI_2_ has not yet been shed, and dC^1^/dC_1_, dP^3^/dP_3_, and dP^4^/dP_4_ are still functional. Permanent teeth that are fully erupted include I^1^/I_1_, P^2^/P_2_, and M^1^/M_1_ (P2 is present on plate B [1). Erupting teeth include I^2^/I_2_ and M^2^/M_2_. Crowns of M^3^/M_3_ are fully formed but lack roots. Crowns of P^3^/P_3_ and P^4^/P_4_ are partially formed, with P^4^/P_4_ notably more developed than P^3^/P_3_. The crown of C^1^ appears to be fully formed, while that of C_1_ is less mineralized. Judging from the stage of crown formation, premolars erupted in the sequence P^2^/P_2_ – P^4^/P_4_ – P^3^/P_3_, as in *Cantius* (Gingerich and Smith, in prep.), *Notharctus* [23, pl. LII: 9], and *Europolemur* [16, pl.III: 3].

Premaxilla: The suture between the two premaxillae is recognizable between the central incisors. The right premaxilla contains two permanent incisors (Figs. 4–5). The bone is almost triangular and has a long caudal suture with the maxilla, as well as a straight, upwardly-directed suture with the premaxilla of the left side. Above there is also a dorsomesial suture with the right nasal. The bone reaches distally to above upper dC^1^.

Maxilla: The bone forms a large part of the face. It contains the canine, two deciduous premolars, P^2^ and three molars (Fig. 4). The maxilla is very flattened and damaged and hard to distinguish from the other bones. The anterior border is located above the precanine diastema. Its suture with the premaxilla is steep and curving caudally into the suture with the nasal. There might be a median process of the maxilla dorsal to the lachrymal as seen in *Lemur*, but this cannot be decided from the X-ray photographs or CT scans. In the intraorbital part of the maxilla, there is a large intraorbital foramen. The mesial opening of the infraorbital channel is very small and situated above the metacone of dP^4^.

Lachrymal: The lachrymal bone is crushed. There seems to be a substantial facial part, but most of the bone lies within the orbit. The lachrymal foramen is not visible.

Frontal: The frontal bone forms the medial and upper half of the posterior border of the orbit. Mesially, it has a suture with the nasal and lachrymal. There is a well-defined ethmoidal foramen. The processus jugalis is robust and meets the processus frontalis of the jugal halfway. Together the two bones form the postorbital bar.

Jugal: The mesiodorsal beginning of the zygomatic arch as well as the ventral border of the orbit is situated above the metacone of dP^4^. The zygomatic arch is mesially low and slender. The jugal size increases considerably distally until the divergence of the processus frontalis. Behind this the jugal narrows to about half of its former height. This is also the width of the postorbital bar.

Squamosum: The bone forms the posterior half of the zygomatic arch and ends caudally in the fossa glenoidalis.

#### Auditory region

Squamosum: Caudally of the rather massive processus postglenoidalis there is a deep porus acusticus, which is not surrounded by an external meatus. The squamosum forms the dorsal roof of the meatus.

Petrosum: The bulla tympanica has completely collapsed. However, the posterior and dorsal part is visible. The bulla of the left side is preserved on plate B, where the dorsal half of the annulus tympanicus is clearly seen on the X-ray photograph [1: fig. 5].

#### Braincase

Part of the left parietal and frontal is visible above the well exposed sutura sagittalis. Because of compaction, the skull appears higher than it was originally. A crista sagittalis was not developed. The rather voluminous braincase ends distally at the crista nuchalis. Caudoventrally, the *in situ* planum nuchale is turned up and crushed.

The following bones form the dorsal and lateral parts of the braincase:

Frontal: As usual, the bone forms the mesial part of the braincase.

Parietal: The bone makes up most of the lateral side of the braincase. It is both deep and wide. Mesially, the parietal meets the frontal bone and caudally it has a long suture with the dorsal part of the occipital. It ends posterolaterally at the nuchal crest.

Occipital: The dorsal extension of the occipital bone (protuberantia occipitalis externa) is wedged between the parietals as a triangular plate.

On the caudal end of the skull, the dorsal rim of the foramen magnum is visible. The atlas is visible to the right of the foramen magnum, pressed against the occipital plane.

#### Lower jaw

The right ramus mandibularis is exposed laterally, with the teeth visible in buccal view. In contrast to adapid skulls [28], its height increases mesially, but not as much as it seems on its left counterpart [1: 293, fig. 4]. In addition, the mesial outline of the mandible is not as steep as it is on the left side (plate B). Both may result from damage during preparation. The micro-CT shows that the symphysis was fused ventrally but still open dorsally, due to the juvenile age of the individual (see below). The angular area increases caudally, where it extends into a well developed, caudally-protruding processus angularis. Some flat bony fragments located ventral and caudal to the processus angularis seem to belong to the hyoids. The processus articularis is still articulated with the fossa glenoidalis, which is situated about 6 mm above the occlusal surface of the mandibular cheek teeth. The coronoid process appears dorsal to the arcus zygomaticus, but it is not fully exposed making description impossible. There is only one foramen mentale appearing below P_2_ in the middle of the corpus.

#### Dentition

The dentition of *Darwinius masillae* shows the holotype to be a juvenile, and imaging reveals a host of developing teeth within the face and jaw (Fig. 5). Much of the face preserves natural occlusion of upper and lower teeth. Studies of higher primates show that teeth generally begin eruption sometime after roots begin to mineralize, emerging through bone and gum before roots are complete [28]. In this light, images of *Darwinius* crown and root development reveal a coherent, readable pattern, in which we see: (1) fainter, less dense deciduous crowns with long roots; (2) developing permanent molars with densely mineralized crowns and incomplete roots; and (3) mineralizing crowns of the replacing permanent teeth (I^1^/I_1_-P^4^/P_4_), largely, but not entirely, buried within the face. Basically, the entire permanent dentition was mineralizing while the deciduous dentition had only begun to be shed.

Deciduous teeth: In the mandible, it appears that the central deciduous incisors (dI_1_) have been shed and replaced. Much smaller second deciduous incisors (dI_2_) remain in the mandible, on right and left sides. We cannot positively identify any upper deciduous incisors, which may have been shed. Clearly, upper and lower deciduous canines are in place. All four deciduous third and fourth premolars (dP^3^/dP_3_ and dP^4^/dP_4_) are erupted and in occlusion. All the deciduous teeth have long roots, consistent with circumnatal emergence. At the second premolar position we see only a single tooth generation in the mandible and maxilla, and, after more extensive comparison, conclude that dP_2_ was probably shed at an early age.

Permanent molars: All three permanent molars can be seen in the dentary. The first permanent molar in the dentary, M_1_, is fully erupted, occluding in normal position with M^1^. The long, but open roots of M_1_ suggest that it was probably erupted for some time (weeks or possibly months). The mandibular second molar, M_2_, is just erupting, and its roots are less developed. The upper second molar, M^2^, is displaced but lacks sufficient root development for eruption. Third molars, M^3^/M_3_, had no roots mineralized, and these crowns were probably still covered by soft tissues.

Replacement teeth: The first permanent incisor is the most advanced of the replacement teeth; this tooth is fully erupted with root length mineralized perhaps ⅔ or ¾ of final adult length. The tooth labeled I_1_ is permanent because it is much larger than dI_2_ and it has a denser crown. Development of I_2_ is well underway, but it is significantly behind I_1_.

In the premaxilla, we can see four incisor teeth. The right side is clearest: here, the I^1^ (with its labial edge slightly broken) is erupted, with a long root (¾ or more mineralized). The more caniniform right I^2^ shows root development of about ⅔. Radiographs also show a well developed incisor from the left side that is more difficult to identify (it may be I^1^ or possibly I^2^; one of these teeth is missing in either case). Maxillary permanent incisors were at or near emergence.

The developing lower canine crown, C_1_, is substantial, but probably no more than half its eventual size. The massive upper permanent canine crown is probably caught at its maximum width, as mineralization was just outlining flanges at the base of the crown, indicating that a wide but not extremely tall crown was forming. The second premolar is represented by a tiny maxillary tooth, P^2^, on plate B, and a small mandibular tooth, P_2_, on both plates A and B. The mandibular tooth has a more densely mineralized crown, casting a denser shadow on radiographs and allying it with other permanent teeth. Root development is long and clearly advanced over that of the remaining permanent premolars. The crown of P_4_ is less than ½ formed, but noticeably advanced over that of P_3_; crowns of P^4^ and P^3^ can be identified in radiographs, with P^4^ again much advanced over P^3^.

#### Molar morphology

**Figure 6:**
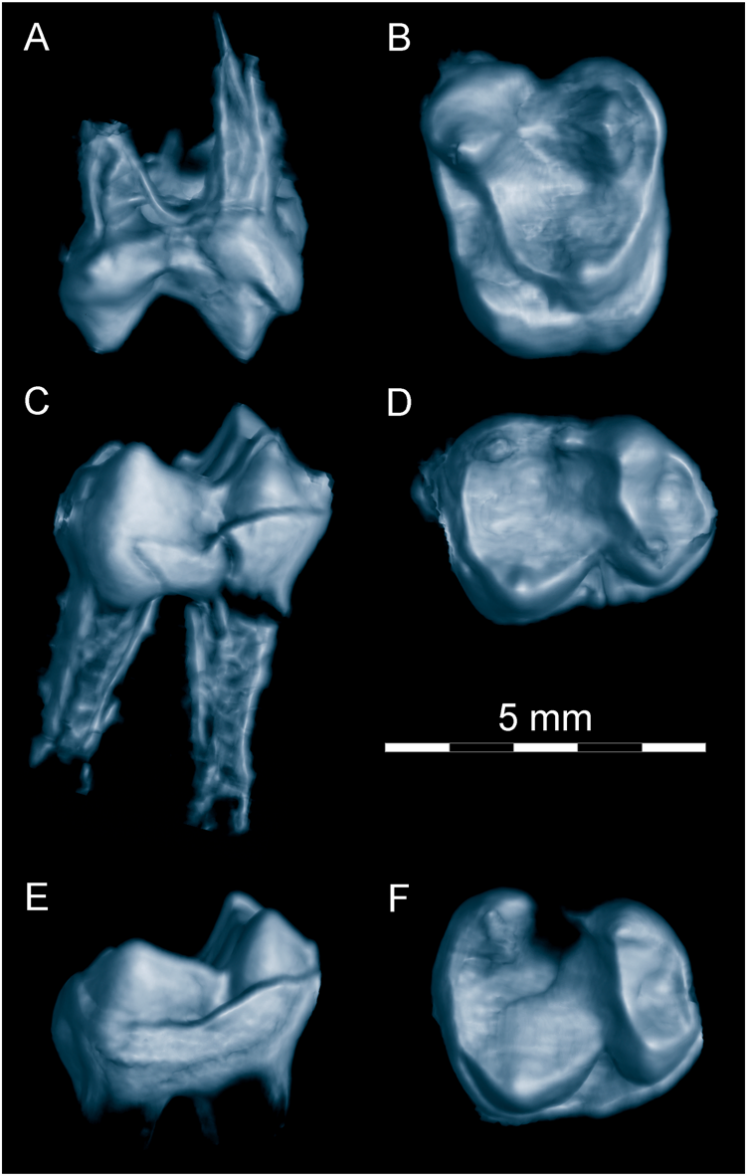
Micro-CT reconstructions of molar teeth of *Darwinius masillae*, new genus and species. Tooth crowns shown here were extracted digitally to show the entire crown for teeth that are only partially exposed in Plate A (see Fig. 5). (A–B)— right M^1^, in buccal and occlusal view. (C–D)— right M_1_, in buccal and occlusal view. (E–F)— right M_2_, in buccal and occlusal view. Note the absence of a mesostyle on M^1^, and the presence of a hypocone on the broad lingual cingulum of this tooth. Note too the absence of a distinct paraconid and hypoconulid on M_1–2_, and the very broad talonid on M_2_. Molars of *Darwinius masillae* are distinct in morphology and intermediate in size between those of contemporary species of *Periconodon* and *Europolemur*.

Little can be seen of the crowns of the molars in either plate A (Fig. 5) or plate B. However, we have succeeded in extracting three molars using micro-CT and graphic reconstruction (Fig. 6).

The crown of M^1^ is subrectangular in occlusal outline, with a prominent protocone, paracone, and metacone well spaced on the crown. There is a well-developed hypocone developed on a broad lingual cingulum, but a pericone, if present, was weakly developed (Fig. 6A–B). This tooth has the classic simplicity of cercamoniine upper molars. Roots are relatively well developed, which is consistent with its early eruption. Measurements are listed in Table 2.

The crown of M_1_ is relatively long and narrow (Fig. 6C–D). There is no distinct paraconid, but a looping paracristid encloses a basined trigonid. The protoconid and metaconid are well developed on the trigonid, followed by a distinct hypoconid and entoconid on the talonid. There is no hypoconulid, but a well developed metastylid distally from the metaconid. The talonid of M_1_ is distinctly broader than the trigonid, but less broad than the talonid on M_2_. The cristid oblique or prehypocristid runs mesiolingually toward the notch in the postprotocristid but then turns abruptly to join the protoconid. There is a narrow cingulid bordering the lingual side of the tooth. Measurements are listed in Table 2.

The crown of M_2_ is shorter than that of M_1_, with a broader trigonid and a much broader talonid (Fig. 6E–F). The trigonid is short anteroposteriorly. It lacks a paraconid, and again has a looping paracristid enclosing a shallowly basined trigonid. The protoconid and metaconid are well developed on the trigonid, and again they are followed by a distinct hypoconid and entoconid on the talonid. There is neither a hypoconulid nor a metastylid. The lingual cingulid is more pronounced than that on M_1_. As on M_1_, there is a distinct cristid oblique that ends near the base of the protoconid. Measurements are listed in Table 2 and Appendix S1.

#### Vertebral column

**Figure 7:**
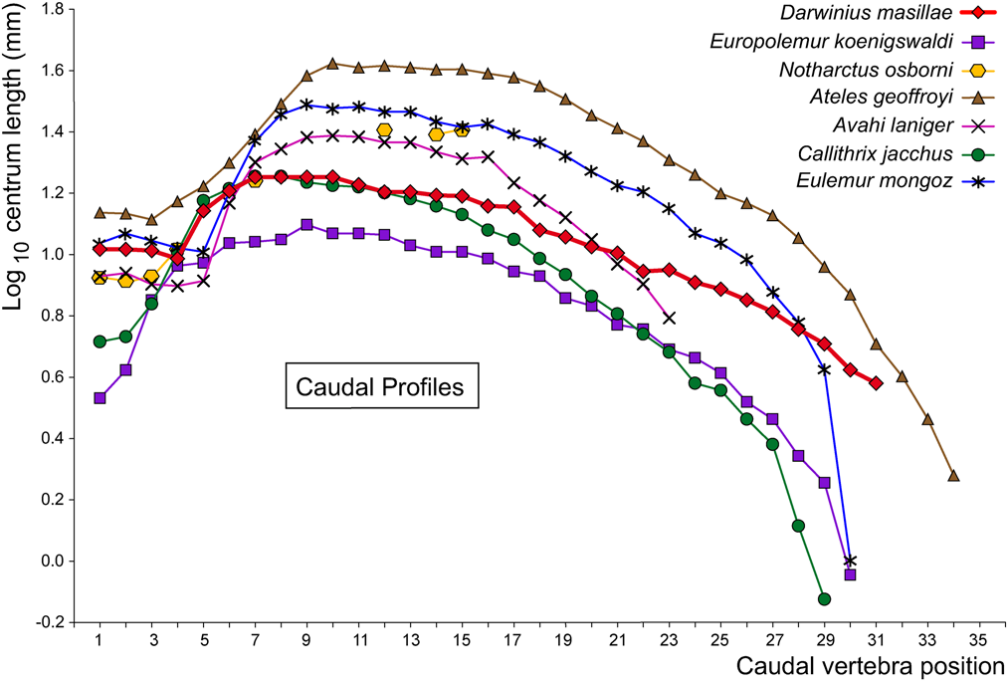
Length profile for caudal vertebrae of *Darwinius masillae*, new genus and species, compared to those of other primates. Measurements of *Darwinius* were taken from plate A (Fig. 1). *Darwinius*, *Europolemur*, and *Notharctus* are Eocene adapoids; *Ateles* and *Callithrix* are extant Ceboidea; and *Avahi* and *Eulemur* are extant Lemuroidea. Measurements of comparative specimens are from [23], [38], with new measurements added for *Avahi*. Note that the profile for *Darwinius* is flatter (rises less high and declines less rapidly) than that for any of the comparative specimens.

(Figs. 1–2, 7, S3, and measurements in Appendix S1). The vertebral column is complete, although laterally compressed and, in part, crushed. Altogether it comprises 7 cervical, 11 thoracic, 7 lumbar, 3 sacral, and 31 caudal vertebrae. The whole vertebral column, from the proximal end of the atlas to the end of the last caudal vertebra, measures ca. 53 cm (Table 2). Together with a basal length of the skull of about 5 cm, this results in a total skeleton length of ca. 58 cm, whereas the head and body length is ca. 24 cm without the tail.

The atlas is broken and incomplete. It is attached to the planum nuchale of the cranium. The left wing of the atlas is crushed, whereas the right wing is seen in dorsal view, with a well-developed foramen vertebrale laterale. The lateral surface of the axis is visible in plate A, however the prominent processus spinalis is crushed. C3–C5 are visible in lateral view. Their processus spinales are only partially exposed, whereas their processus transversi are clearly visible. Caudally in the cervical series, the processus transversi become more and more expanded. C6 is crushed, whereas the right scapula covers C7.

By including the first and second thoracic vertebrae, which are hidden below the right scapula, 11 thoracic vertebrae are present although their exact number is difficult to determine and therefore somewhat ambiguous. Whereas T3–T5 are laterally exposed, T6–T8 have rotated around their long axis so that they are seen in dorsal aspect, while T9–T11 are visible laterally. There is no diaphragmatic vertebra, because even the processus spinalis of T11 is slightly but clearly dipping caudally. The ribs are not well preserved. Most of their cartilaginous parts exist only as natural casts. The right humerus mostly covers the sternum.

Caudal to the thoracics are 7 lumbar vertebrae. They are comparatively massive and display cranially oriented transverse processes, which become more and more expanded caudally. No spinal processes are evident on L1–L3, but L4 carries a rather small process slightly dipping caudally. The spinal process of the lumbar vertebrae becomes somewhat larger caudally and dips more in this direction. The os sacrum comprises 3 vertebrae, S1–S3, the most proximal one of which is damaged.

Altogether, there are 31 caudal vertebrae but the last one ends fragmented at a fault. So there may have been one or two more. The 3 most proximal are comparatively short and display strong transverse processes that become weaker more distally in the series. The last transverse process is developed on Ca4, which is already considerably longer and shows only a small processus transversus at its caudal end. All following vertebrae have no processus transversi.

In *D. masillae* the dorsal vertebral column shown on plate A is gently curved (that of plate B is fake) and the tail is only slightly curved. The length profile of the proximal half of caudal vertebrae is close to that of living *Callithrix jacchus*, while more distally *D. masillae* has much longer vertebrae. Altogether, the tail is much longer than that referred to *Europolemur koenigswaldi* (Fig. 7). In *D. masillae*, the length profile of Ca8–Ca20 differs from that of the living *Avahi laniger*, even more so from *Eulemur mongoz*, and considerably from *Ateles geoffroyi*. Clearly, *Darwinius* did not have a prehensile tail. The tail was presumably used primarily for balance, and possibly for steering while leaping. Its soft body contours are incomplete. Therefore, it is impossible to decide whether it was bushy or not.

#### Shoulder girdle and forelimb

**Figure 8:**
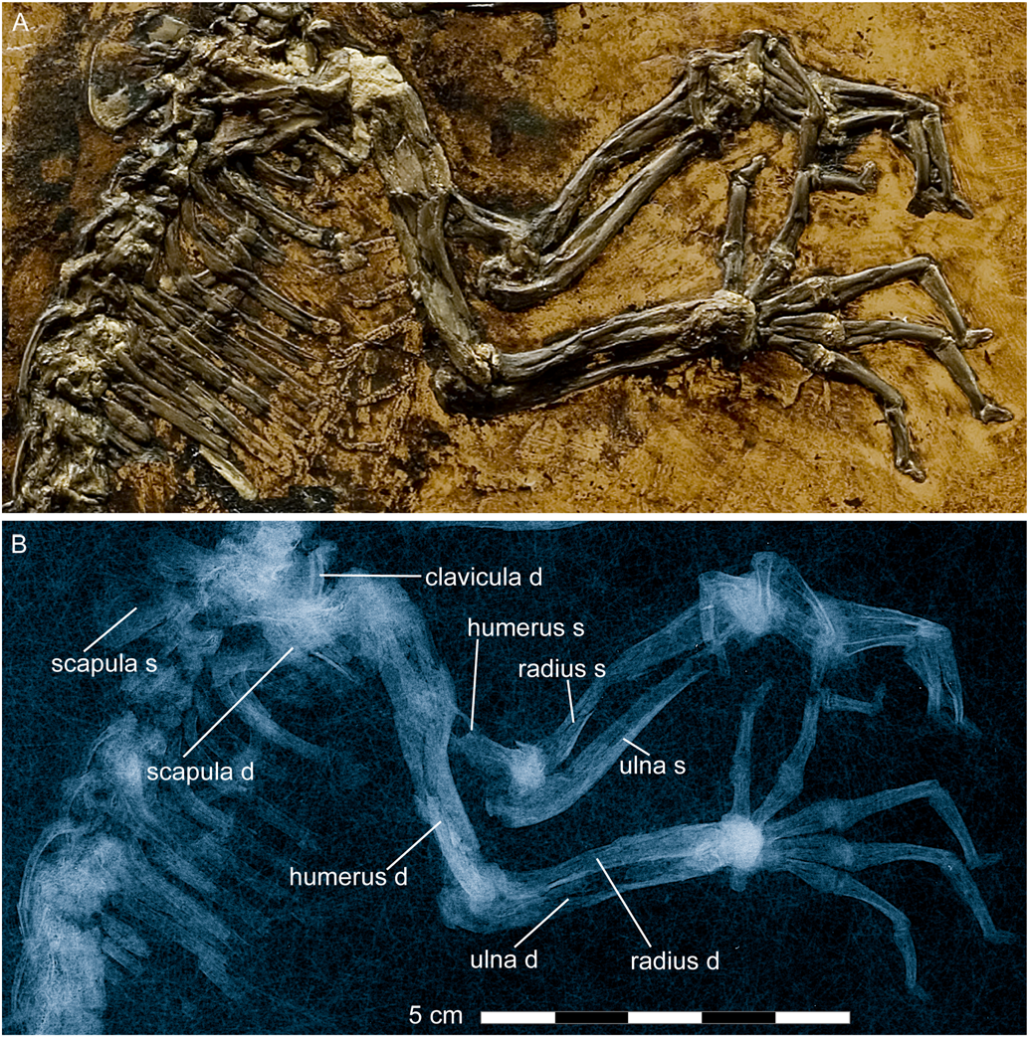
Shoulder girdle and forelimb of *Darwinius masillae*, new genus and species. Photograph (A) and X-ray image (B) show the specimen preserved on plate A (Fig. 1). Note excrescence at the distal end of the right forearm, and a fracture of the basal phalanx of the left pollex (details are shown in Fig. 9).

**Figure 9:**
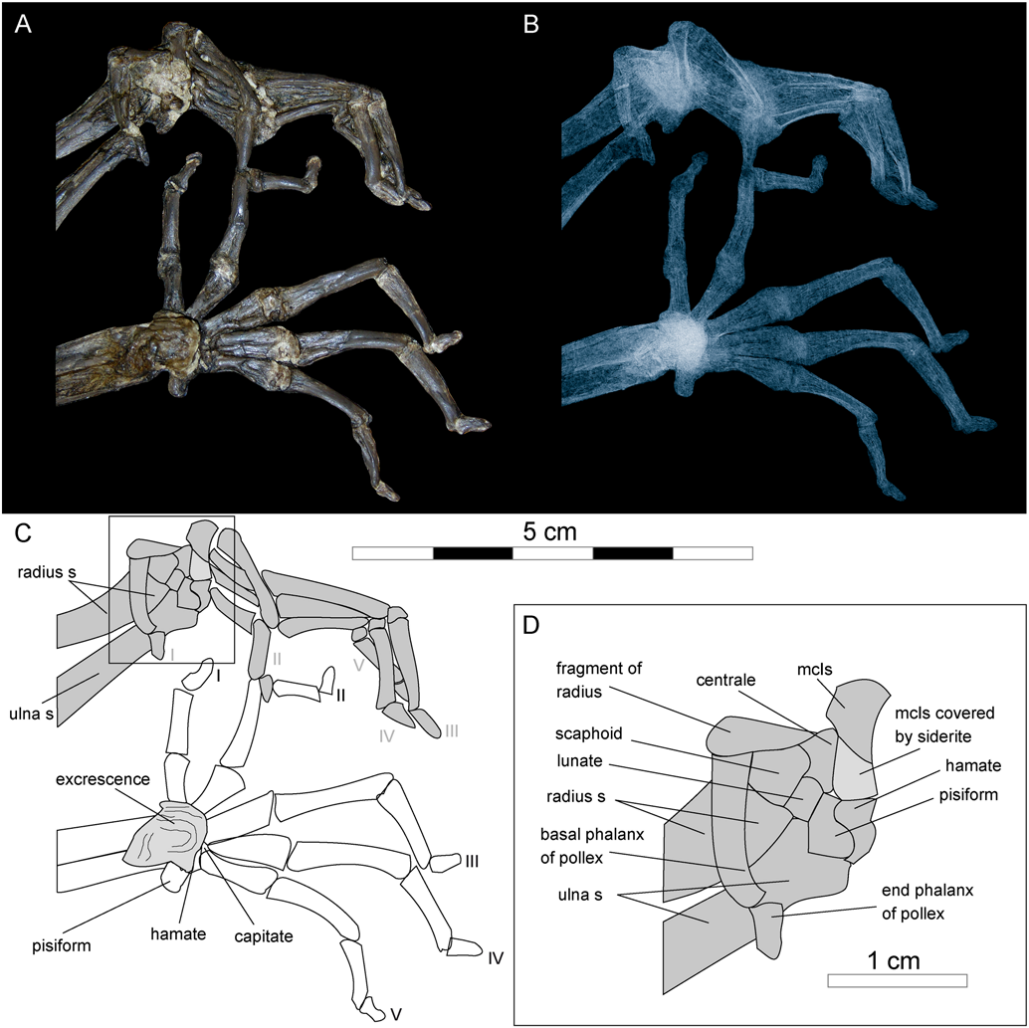
Hands and wrist of *Darwinius masillae*, new genus and species. Photograph (A) and X-ray image (B) show the specimen preserved on plate A (Fig. 1). (C)— explanatory drawing, where I–V represent digits one to five. (D)— Inset interpretive drawing of the left wrist (box in C).

(Figs. 8–9, S4, and measurements in Appendix S1). The right scapula represents most of the shoulder girdle (Fig. 8). Its dorsal part is heavily crushed. The crista scapulae passes proximocranially into a rather expansive processus hamatus for articulation with the clavicle. The left scapula appears dorsal to the vertebral column and its dorsal part can be viewed medially. The processus hamatus is curved in a craniodorsal direction, more so than in *Notharctus osborni*, while the caudal extension of the margo costalis dorsal to the collum is not as expressed. In *Eulemur mongoz*, *Varecia variegata*, *Avahi laniger*, and *Loris* sp., such a caudal extension of the margo costalis is totally missing, and the same holds for *Callithrix jacchus* and *Cercopithecus neglectus*. Dorsally, the crista scapulae reaches the margo vertebralis of the scapular blade. The facies supra spinam is evidently much smaller than the facies infra spinam. A fragment of the clavicle can be seen dorsal to the processus hamatus of the right scapula, but no details are observable.

The right humerus is well exposed in lateral view. Only the distal part of the left humerus can be seen, in medial view. Both are articulated with their respective forelimbs. Proximally, the epiphyseal suture is still present, although nothing can be said about the proximal epiphysis because it is completely obscured by siderite. Here, as elsewhere on the skeleton, siderite formed as a concretion around decomposing cartilage. The crista deltoidea of the humerus is well developed and runs up to the middle of the distal diaphysis. A crista brachiolateralis (crista epicondyli lateralis) is visible distally, and this expands as is seen typically in prosimians. It is not as broad as that of *Notharctus osborni*, and it is more like that seen in *Eulemur mongoz*, *Varecia variegata*, and *Avahi laniger*. Mediocaudally, where the left humerus is close to the trochlea, a foramen entepicondyloideum is well developed as common to many mammals as well as primitive anthropoids such as platyrrhine primates.

The trochleae of both humeri are in articulation so no details are visible.

The ulna and radius are completely separated, as is typical for primates. The forearm is unusually short, being about the same length as the humerus. This is the case in *Varecia variegata*, *Callithrix jacchus* and *Cercopithecus neglectus*, whereas the forearm becomes proportionally longer in the series *Eulemur mongoz*, *Notharctus osborni*, *Avahi laniger*, and especially *Godinotia neglecta* from Geiseltal. The right forearm of *Darwinius* is preserved in pronation, so that the radius is exposed from the lateral side and the ulna is viewed medially. The left forearm, however, is preserved in supination, so that the radius and ulna are both seen from the medial side. The ulna is more robust proximally, while the radius is more robust distally. Proximally the caudal outline of the ulna curves cranially, while distally the radius curves in a caudal direction. The processus olecrani is short but high when compared with *Lemur*.

The left ulna has a well developed processus anconaeus, and the incisura semilunaris is deep. The distal end of the left forearm is still articulated with the carpus, whereas that of the right forearm lies on top of the carpus. In both cases, articular facets are not discernible. Of special interest is a substantial excrescence that inflates the distal ends of the right ulna and radius, causing them to be secondarily fused (Figs. 8–9). The excrescence is of bone and differs in both color and structure from the bright yellow siderite below and between bone fragments. Clearly, on the right arm the callus covered and fused the carpus. Evidently the animal suffered a fracture at the distal end of the right forearm. The latter covers the carpus, so that only the hamate and its articular facets for metacarpal V and a small part of the capitate are visible. In an X-ray the proximal articulation of the right metacarpal I with the trapezium is not clearly visible. However, the mediolateral extension of the proximal epiphysis of metacarpal I suggests that this was a saddle-shaped rather than a ball and socket articulation. This is confirmed by the left carpus.

The left carpus is proximally exposed from its palmar side. Left metacarpal I is proximally disarticulated, exposing part of the articular facet for articulation with the trapezium. This articulation is clearly saddle-shaped, indicating beyond doubt that the thumb was opposable. The proximal carpals include a transversally oriented pisiform that articulated originally with the ulna proximally and the hamate distally. The rather small lunate articulates proximally with the radius and with the ulna, medially with the scaphoid, and distally with the centrale (Fig. 9). In *Darwinius* the arrangement of the carpals corresponds to that known for *Europolemur* and *Notharctus* [17], [30]. It differs from the arrangement in *Adapis*, where the lunate is excluded from any contact with the centrale [27], [31]. Metacarpal II lies across the distal metacarpals, exposing its dorsal side. Proximally it is disarticulated, so that the face of its saddle-shaped articulation with the trapezoid is exposed. The distal end of metacarpal I is covered by siderite, and this is more or less hidden below the pisiform and the hamate (Fig. 9). Metacarpals III–V are all seen in palmar view, but their proximal articulations are mostly hidden by metacarpal II. Metacarpal V, displays much of a saddle-shaped articulation with the hamate. With the exception of the pollex, all of the basal phalanges are very long: the longest being digit III, followed by digits IV and V. Digit II is a little shorter than digit V, and the shortest digit is that of the thumb.

The articulated basal and terminal phalanges of the pollex lie across the distal ends of the radius and ulna. The distal end of the basal phalanx appears to be somewhat deformed, being bent lateropalmarly. It is exposed in lateral aspect. An X-ray (Fig. 9) shows a transverse fracture of the midshaft of the basal phalanx.

The terminal phalanx of the pollex, on the lateral side of the ulna, is scutiform in dorsal view. The basal phalanx of the second digit is completely exposed from its medial side, and its distal half covers most of the distal ends of metacarpal II–V. The complete basal phalanx of digit III is seen in medial view. It articulates proximally with metacarpal III, as do metacarpals IV–V with digits IV and V, respectively. Whereas the basal phalanx of digit IV is exposed palmarly, that of digit V is exposed laterally. In a distal direction, the basal phalanges of digits III and V come so closely together that the distal end of the basal phalanx of digit IV is almost completely covered by them. The intermediate phalanges of digits III–V are all exposed in medial view, and digit IV is seen crossing over the diaphysis of the intermediate phalanx of digit V. The terminal phalanx of digit I is exposed from the dorsal side, whereas those of digits II–IV, are seen in palmarolateral view. The terminal phalanx of digit V is exposed between the intermediate phalanges of digits III and IV. All are scutiform, and hence were nail-bearing.

On the right hand, all metacarpals and most phalanges are exposed in dorsal view. Only the phalanges of digit V are turned progressively so that the terminal phalanx is completely exposed in palmar view. The lengths of the basal and middle phalanges of digits II–V are remarkable and resemble those of the modern *Lemur*, whereas the metacarpals are much shorter. The latter, as well as the basal and middle phalanges, especially the latter distally, are slightly bent palmarly. In contrast to the hallux, the pollex is rather small and short. All terminal phalanges of the right hand clearly bore nails.

All in all, the hand of *Darwinius masillae* is much stouter than that of *Europolemur kelleri*, *Godinotia neglecta*, or *Notharctus osborni*, even though there is not a great difference between the lengths of the metacarpals and basal phalanges. *Darwinius* together with *Notharctus* and *Europolemur*, but not *Adapis*, have a hand similar to those of living galagines. The function of the hand is evidently not correlated particularly well with locomotor type [32: 273], although it must constrain the size of branches the hand could grip. The functional significance of mesaxony in primate hands and feet, which *Darwinius* shares with *Europolemur* and living anthropoids, is not clear.

#### Pelvis and posterior limb

**Figure 10:**
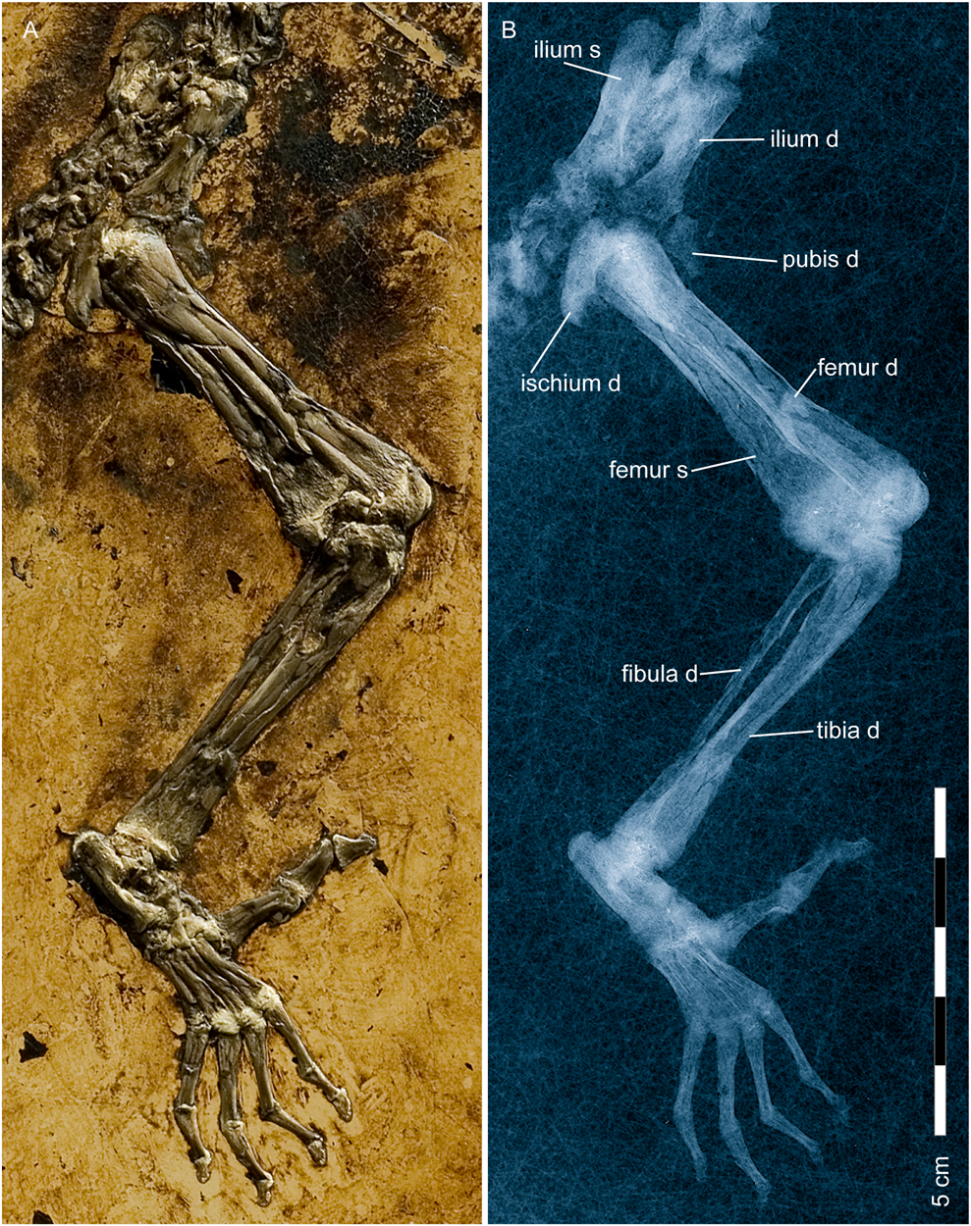
Pelvis and hind limb of *Darwinius masillae*, new genus and species. Photograph (A) and X-ray image (B) show the specimen preserved on plate A (Fig. 1). Note the large opposable hallux. Hind limb proportions are compared to those of other primates in see also Figure S5, and an explanatory drawing is provided in Figure 11.

**Figure 11:**
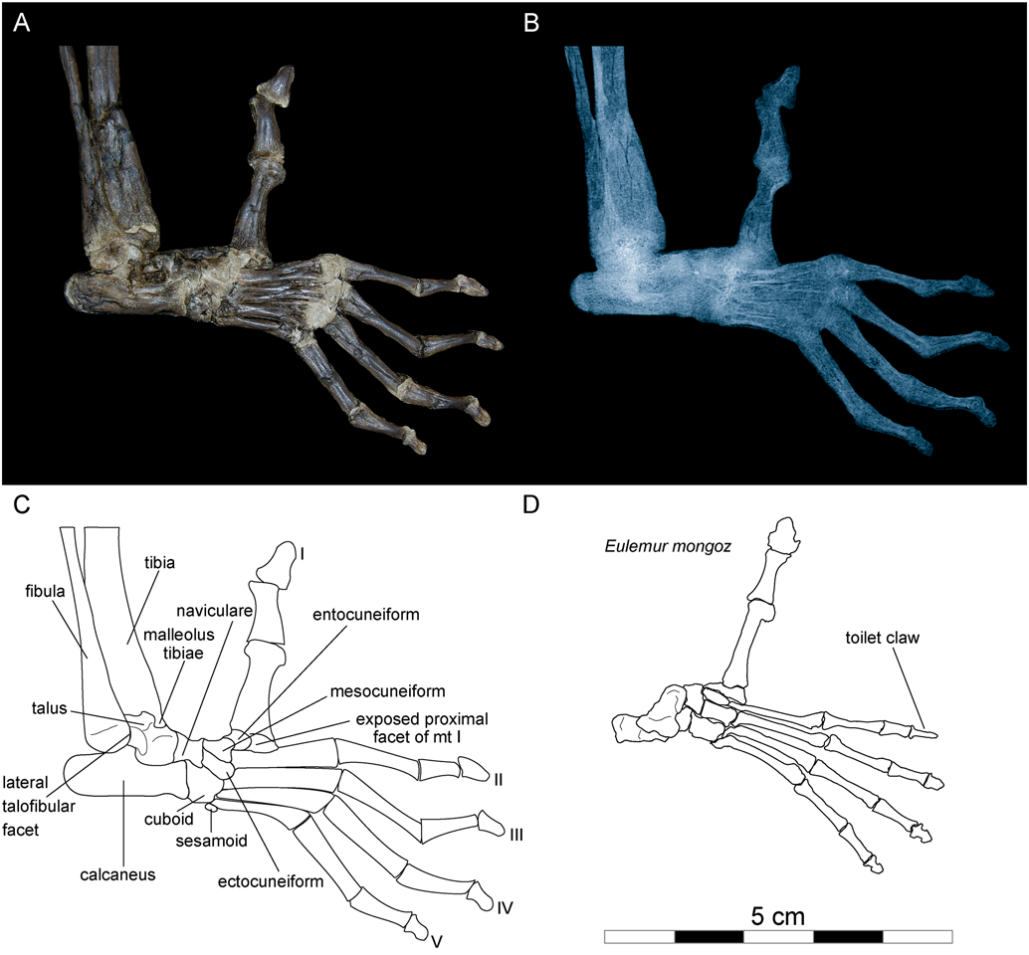
Right foot of *Darwinius masillae*, new genus and species. Photograph (A) and X-ray image (B) show the specimen preserved on plate A (Fig. 1). (C)— explanatory drawing. (D)— drawing of foot of *Eulemur mongoz* for comparison. Note the large opposable hallux, and absence of a grooming claw on digit II in *Darwinius*.

(Figs. 10–11, S5, and measurements in Appendix S1). The right side of the pelvis is visible in lateral view, with the ilium, pubis and ischium still not fused (Fig. 10). The os sacrum and vertebral column cover most of the left side. The articular surfaces of the acetabulum and the caput femoris cannot be seen, but the latter is surrounded by the ilium craniodorsally and the pubis cranioventrally, and by the ischium posteriorly. Consequently, the foramen obturatum is completely hidden. The iliac blade is narrow and extends craniocaudally as in prosimian primates, although such morphology also occurs in *Callithrix jacchus*. It is as narrow as in *Loris*, and clearly narrower than in *Cercopithecus neglectus*. The tuber sacrale is situated dorsomedially near the middle of the ilium. The crista iliaca is short and cranially convex. Details of the pubis are restricted to the cranially directed pecten. Compared with *Lemur*, the tuber ischiadicum is rather weak.

The proximal part of the left femur is mostly covered by that belonging to the right side, which is laterally exposed. Compared with the caput, the neck of the femur is very short and the trochanter major is very low as in *Notharctus osborni*. The trochanter major is higher in *Eulemur mongoz*, *Varecia variegata*, *Avahi laniger*, and particularly *Cercopithecus neglectus*. All growth sutures are still open and unfused. The right patella is exposed laterally. The distal end of the left femur is slightly shifted cranially so that the distal part becomes visible.

The left lower limb is missing except for a short proximal fragment beyond the knee joint. Most of the left lower leg is preserved on plate B, although its distal end and the foot are also missing on plate B. Most probably, this part was lost during excavation of the specimen as there are no signs of damage or bite marks on the adjacent bones. The lower leg and foot of the right limb are completely preserved. The tibia is seen in lateral view and the fibula is exposed mainly from its cranial side. Both lie parallel to each other and are not fused. Proximally as well as distally, growth sutures are still visible. The crista tibiae is not well defined, and the proximal end of the tibia is slightly bent caudally as in *Lemur*, but not to the extent as that of *Godinotia neglecta* from Geiseltal [22: 58–60, fig. 2.18].

The tarsus is exposed in laterocranial view (Fig. 10), with the processus coracoideus situated dorsally from behind the middle of the calcaneum as in *Lemur* and *Europolemur kelleri* [13: 70–71]. This differs considerably from omomyids and even more so from *Tarsius*, in which the part of the calcaneum distal to the processus coracoideus is extremely elongated, while it is much shorter in anthropoids. Hence it appears plesiomorphic for prosimians. Except for its smaller size, the tarsus of *Darwinius*, seen in lateral view, resembles that of *Adapis parisiensis* figured by Decker & Szalay [33: fig. 3]. The talofibular facet is steep and the peroneal tubercle is rather small and sharply angled [33] which is unlike that seen in adapids, *Lemur*, *Hapalemur*, and other lemuriforms, and is more like that in haplorhines (see [34], [35] for discussion). Unfortunately, the groove for the flexor fibularis cannot be seen while only a small part of the talotibial facet is exposed. These two characters, together with the shape of the talofibular facet, form the talar morphology shared by known Eocene adapiforms with lemuriforms and lorisiforms [33], [35]. The steep fibular facet on the talus or astragalus alone is not a synapomorphy for anthropoids because it also occurs in outgroups such as Scandentia, Dermoptera and Plesiadapiformes. Among primates it is, however, a haplorhine apomorphy[35], [37], and its presence in *Darwinius* supports taxonomic and phylogenetic classification with haplorhines rather than strepsirrhines (Table 3).

**Table 3:** Interpretation of morphology of *Darwinius masillae* in comparison to characteristics distinguishing extant strepsirrhine and haplorhine primates.

	Anatomical/morphological characteristic	Lem	Lor	Tar	Ceb	Cer	Hom	ref	Primitive or derived	*Darwinius masillae*	Interpretation
	**Strepsirhini**										
1	Moist nose with median cleft in upper lip	X	X					74: p.24	Primitive	N/A	—
2	Jacobson's vomeronasal organ	X	X	X	X			74: p.24	Primitive	N/A	—
3	Sphenoidal recess in nasal cavity	X	X					74: p.23	Primitive	N/A	—
4	Reflecting tapetum lucidum in eye	X	X					74: p.82	Derived?	N/A	—
5	Small brain and braincase	X	X	X				74: p.82	Primitive	Present	—
6	Brain with relatively large olfactory bulbs	X	X	X				74: p.20	Primitive	N/A	—
7	Stapedial/pharyngeal blood supply to brain	X	X					74: p.22	Derived?	N/A	—
8	Cranium with long rostrum	X	X					74: p.15	Primitive	Absent	—
9	Shallow mandibular ramus	X	X					74: p.15	Primitive	Absent	—
10	Open metopic suture between frontal bones	X	X	X				74: p.15	Primitive	Present	—
11	Postorbital bar without postorbital closure	X	X					74: p.82	Primitive	Present	—
12	Ectotympanic free or in lateral wall	X	X		X			74: p.28	Primitive	Present	—
13	Open mandibular symphysis	X	X	X				74: p.13	Primitive	Partial	—
14	Procumbent to vertical pointed incisors	X	X	X				74: p.15	Primitive	Absent	—
15	Tooth comb of lower incisors-canines	X	X					74: p.82	Derived	Absent	—
16	Non-dimorphic canine teeth	X	X	X				87	Primitive	N/A	—
17	Upper molars quadrate with hypocone cusp	X	X		X	X	X	75: p.53	Derived	Present	Indet.
18	Premolar P_4_ molarized	X	X					75: p.53	Derived?	N/A	—
19	Lower molars quadrate w. reduced paraconid	X	X		X	X	X	75: p.53	Derived	Present	Indet.
20	Capitate (os magnum) laterally compressed	X	X					75: P.51	Derived	N/A	—
21	Sloping fibular facet on astragalus or talus	X	X					74: p.82	Primitive?	Absent	—
22	‘Tarsi-fulcrumating’ pes with long tarsals	X	X	X				75: p.40	Derived	Absent	—
23	Mediolaterally-compressed mesocuneiform	X	X					75: p.52	Derived	Absent	—
24	Pes with fourth toe longest	X	X	X				75: p.40	Derived	Absent	—
25	Grooming claw on pedal digit II	X	X	X				74: p.82	Primitive	Absent	—
26	Two or more pairs of mammary glands	X	X	X				74: p.82	Primitive	N/A	—
27	Bicornate uterus	X	X	X				74: p.83	Primitive	N/A	—
28	Epitheliochorial placenta	X	X					74: p.83	Primitive	N/A	—
29	More precocial (more teeth at birth)	X	X	X	X			50, 88	Primitive	N/A	—
30	Lack of SINE human Alu transpositions	X	X					89	Primitive	N/A	—
	**Haplorhini**										
1	Dry nose and continuous upper lip			X	X	X	X	74: p.24	Derived	N/A	—
2	Loss of Jacobson's vomeronasal organ					X	X	74: p.24	Derived	N/A	—
3	Sphenoidal recess greatly reduced			X	X	X	X	74: p.23	Derived	N/A	—
4	Retinal fovea in eye			X	X	X	X	74: p.82	Derived?	N/A	—
5	Larger brain and braincase				X	X	X	74: p.82	Derived	Absent	—
6	Brain with relatively small olfactory bulbs				X	X	X	74: p.20	Derived	N/A	—
7	Promontory arterial blood supply to brain			X	X	X	X	74: p.22	Derived?	N/A	—
8	Cranium with short rostrum			X	X	X	X	74: p.15	Derived	Present	**Synap.**
9	Deep mandibular ramus			X	X	X	X	74: p.15	Derived	Present	**Synap.**
10	Fused metopic suture uniting frontals				X	X	X	74: p.15	Derived	Absent	—
11	Partial to complete postorbital closure			X	X	X	X	74: p.82	Derived	Absent	—
12	Ectotympanic in lateral wall or tubular			X		X	X	74: p.28	Derived	Absent	—
13	Fused mandibular symphysis				X	X	X	74: p.13	Derived	Partial	**Synap.**
14	Vertical spatulate incisors				X	X	X	74: p.82	Derived	Present	**Synap.**
15	Interlocking canine teeth			X	X	X	X	74: p.15	Primitive	Present	—
16	Sexually dimorphic canine teeth				X	X	X	87	Derived	N/A	—
17	Upper molars quadrate with hypocone cusp	X	X		X	X	X	77: p.16	Derived	Present	Indet.
18	Premolar P_4_ simple w. transverse pad-mcd crest				X	X	X	76: p.16	Derived	N/A	—
19	Lower molars quadrate w. reduced paraconid	X	X		X	X	X	76: p.16	Derived	Present	Indet.
20	Capitate (os magnum) uncompressed			X	X	X	X	76: p.16	Primitive	N/A	—
21	Relatively small, steep fibular facet on astragalus			X	X	X	X	74: p.82	Derived	Present	**Synap.**
22	‘Metatarsi-fulcrumating’ pes w. long metatarsals				X	X	X	76: p.15	Primitive	Present	—
23	Non-compressed mesocuneiform			X	X	X	X	76: p.15	Primitive	Present	—
24	Pes with third toe longest				X	X	X	76: p.15	Primitive	Present	—
25	Loss of all grooming claws				X	X	X	74: p.82	Derived	Present	**Synap.**
26	Single pair of mammary glands				X	X	X	74: p.82	Derived	N/A	—
27	Simplex uterus				X	X	X	74: p.83	Derived	N/A	—
28	Hemochorial placenta			X	X	X	X	74: p.83	Derived	N/A	—
29	Less precocial (fewer teeth at birth)					X	X	50	Derived	N/A	—
30	SINE human Alu transpositions C7, C9, C12			X	X	X	X	89	Derived	N/A	—

Abbreviations: *Lem*., Lemuroidea; *Lor*., Lorisoidea; *Tar*., Tarsioidea; *Ceb*., Ceboidea; *Cer*., Cercopithecoidea; *Hom*., Hominoidea; *N/A*, not applicable; *Synap*., synapomorphy.

The cuboid, which is situated between the calcaneum proximally and metatarsals IV–V distally, articulates proximomedially with a remarkable high navicular bone. On the lateral side a sesamoid is visible. Seen from the lateral aspect only, it is not possible to decide whether it has a pivot joint with the calcaneum like most primates. The navicular is situated between the talus proximally and the ecto- and mesocuneiform distally. It is a long bone compared to that in lorisines, indriids and anthropoids [34], and it is more like that of *Hapalemur* and *Eulemur*, although it is not as wide. The naviculocuboid articulation is broad and contiguous with both the ectocuneiform and mesocuneiform facets shaped like those of living lemuriforms and all known notharctines [36]. Proximolaterally, the navicular articulates with the calcaneum. As is the case with *Eulemur*, the entocuneiform is rather deep. Proximally, it articulates with the navicular bone.

By far the strongest of all metatarsals is metatarsal I, as is the entire hallux. Metatarsal I is about twice as thick as metatarsals II–V. As preserved, metatarsal I extends medially almost at right angles to the other metatarsals when viewed dorsally. Proximomedially, part of the articular facet for the entocuneiform can be seen (Fig. 11). Although partially crushed, and covered laterally by the entocuneiform, metatarsal I appears to be saddle-shaped, indicating that the hallux was opposable. The prehensibility of the hallux corresponds with that of the pollex. Little can be said about metatarsals II–V except for their proportions, which are not as slender as in *Lemur*. Metatarsal II articulates proximally mainly with the mesocuneiform, and only laterally also with the ectocuneiform. Proximally, metatarsal III is supported by the ectocuneiform medially and the cuboid laterally.

All phalanges are exposed mainly from their dorsal side, and all are slightly bent plantarly. Morphologically, the basal and intermediate phalanges do not differ very much from those of the manus, although they are somewhat more robust. This difference in robustness is particularly true for digits I and II of the pes, which are much more robust than their counterparts in the manus. Terminal phalanges IV–V are seen from their dorsal aspects, while III and II are seen progressively but slightly dorsomedially. All terminal phalanges are definitely scutiform, and were therefore nail-bearing, although those of digit II and III appear to be rather narrow. The toilet or grooming claw reported on the second digit of *Europolemur kelleri* [13] cannot be identified here. It is also lacking in *Europolemur koenigswaldi* [38]).

### Paleobiology

The presence of a complete skeleton with soft-tissue body contours and contents of the digestive tract brings us close to the paleobiology of the animal's life and death ( the living individual is reconstructed in Fig. S6).

To begin, we can see something of the process of death and burial. Shortly after death, it appears that the body sank to the lake bottom, landing on its back before coming to rest on its side. There are no bite marks on the adjacent bones to indicate activity of a predator or scavenger.

A dark shadow surrounds almost the whole skeleton, incomplete only at the tail. This shadow indicates the former outline of body and fur produced as a result of bacterial activity [39]. The outline shows that massive muscles surrounded the upper parts of the legs, and that the outer ears were small. Mineral growth at the joints of arms and legs obscure this areas from detailed descriptions. This we interpret as siderite, which often surrounds fossils from Messel and is associated with chemical reactions within the sediments in combination with the rotting of carcasses [8]. In this case, siderite concentration at the joints may be related to the presence of cartilage. In front of the two femora, a dark shadow is associated with coarse material interpreted as contents of the digestive tract on plate B [24]. Previous study of the contents of the digestive tract confirmed the presence of leaves and fruit, but no insects, although insects are often preserved at Messel.

The neck is straight and arms and legs are slightly angled, lying almost parallel. The hands and feet show a somewhat unusual appearance for skeletons from Messel, with the left palm face up and parallel to the bedding (Fig. 9). The metacarpals of the third, forth and fifth finger are close together whereas those of the second and especially the first finger are more widely spread. Fingers are well splayed with last phalanges dorsally inflected. The first toe of the right foot is straight but is directed almost 90° to the others suggesting its potential opposability. These postures make clear that the hands and feet of *Darwinius* had long, highly flexible toes and fingers.

### Sex of the *Darwinius* holotype

Male primates commonly preserve a baculum or penis bone [40]. Four specimens of cercamoniine primates are known from Messel that preserve hind limbs. Two of these have a large baculum preserved in association with the hind limbs. Both are *Europolemur kelleri* (HLD ME 7430 and LNK ME 684), and both are clearly male [13], [14]. Two specimens with hind limbs have no baculum. One is *Europolemur koenigswaldi* (SMNK ME 1125a,b), of unknown sex, and the other is the type of *Darwinius masillae* described here. The specimen of *Darwinius* on plate A is so complete and well preserved, and the known bacula of cercamoniines are so large, that a baculum, if present, should be evident either as a preserved bone or as an impression. Lacking evidence of a baculum, we interpret the holotype of *Darwinius masillae* as female.

### Tooth emergence sequence and the pace of life and aging

Sequence of tooth eruption can inform us about other aspects of primate life history. A broad look at tooth formation of *Darwinius* shows that the third molar crowns are well developed, while the deciduous dentition has only begun to shed– a degree of simultaneous tooth development that does not appear in slow growing primates. This pattern is associated with more rapid growth and aging in primates and in some other mammals [41].

Schultz [42] first noted a regular pattern shift between molars and replacement teeth in primates: the slower-growing primates tend to erupt incisors and even premolars before third or even second molars. In *Darwinius* we can distinguish a first set of teeth that emerged before a second set, (**M_1_ M_2_** I_1_ P_2_) (I_2_ **M_3_** C P_4_ P_3_), an order of tooth eruption that characterizes “medium fast” primates with a maximum life span of about 12–20 years.

Outside of living primates, some very rapidly growing mammals erupt all three molars before replacing any deciduous teeth. The tree shrew for example has the sequence **M_1_ M_2_ M_3_** P_2_ I_3_ P_4_ (I_1_ C) P_3_ I_2_ [43]. Fast-living ungulates have similar sequences [40], and the association of eruption sequence and growth rate continues to hold up in primates as more are studied.

### Life stage of *Darwinius*

Eruption of the first permanent molar marks an important transition for primates, that from infant to juvenile [42]. Primates tend to be weaned about this time, especially species with higher-quality diets [44], [45]. The *Darwinius* holotype, with both first and second lower molars erupted, lived past infancy, was weaned, and had started to feed independently before dying.

To evaluate *Darwinius* maturation further, we must choose a model from living primates. The best predictor of growth rate in primates is adult brain size; body weight is a distant second [46]. The best we can do in present circumstances is to choose a model of similar body size and tooth eruption sequence. Among small to medium-sized living primates, the lemurs (e.g., *Lemur, Eulemur*, and *Varecia*) develop and age on a time scale closely similar to that of the New World monkey *Saimiri*. Living lorisoids (*Loris, Galago*) and the single living tarsioid (*Tarsius*) grow and age on a faster time scale, but fewer data are available for detailed comparisons. *Saimiri* is relatively well studied, allowing the best comparison with *Darwinius* ontogeny. Whether this time scale applies, or one that is a step faster, we can begin to integrate the growth and development of different organ systems.

**Figure 12:**
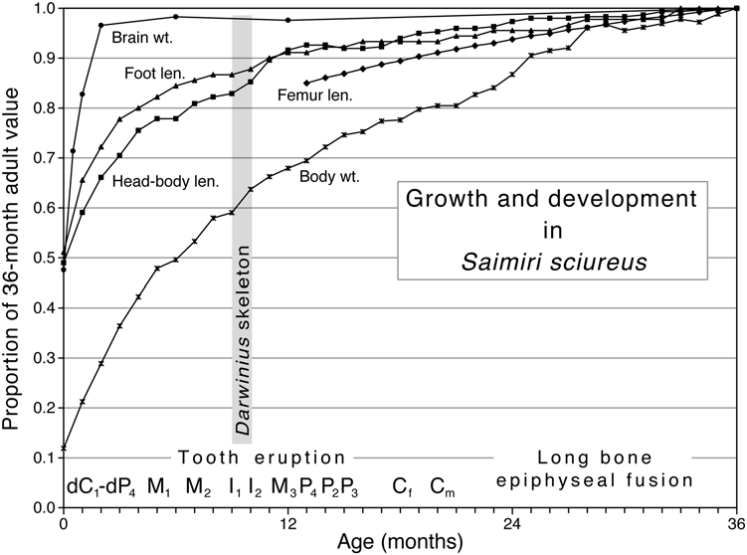
Growth and development in extant squirrel monkey *Saimiri sciureus* as a model for growth expected in *Darwinius masillae*. Stage of tooth eruption indicates that when it died *Darwinius* was about 9–10 months old (gray bar) on a *Saimiri* developmental scale. Growth as a proportion of 36-month adult size is plotted by age in months, with developmental events added for the interval from birth to 36 months along the bottom. *Saimiri* is born with deciduous incisors erupted and quickly completes the deciduous dentition. Note that adult brain weight is achieved rapidly in development; adult foot length, head and body length, and femur length are achieved more slowly; and adult body weight is achieved very slowly. Using *Saimiri* as a model, we estimate that *Darwinius* died at about 60% of its projected adult body weight, 80–85% of its projected adult body and limb length, and 98% of its projected adult brain weight. By this model, *Darwinius* is projected to add 66% (1/0.60) to its weight, 20% to its body and limb length (averaging 1/0.80 and 1/0.85), and 2% to its brain weight (1/0.98). Sources: tooth development, weight, head-body, and foot lengths are from the same captive growth study [79]; other studies give brain weight [80], femur length [81], and long-bone maturation [82].

Figure 12 shows the developmental position of the *Darwinius* holotype in the middle of the period of permanent tooth eruption. As expected from comparison to a range of living primates, major epiphyses remain open.

If *Darwinius* grew on a *Sairmiri* time scale, the holotype individual died at ca. 9–10 months of age. We expect that she would have begun to mature sexually as she neared her third year; with incremental growth possible until about 3 years of age. *Saimiri* females begin to reproduce as early as 30 months, but since they are strictly seasonal breeders in the wild [47], a first birth at 36 months seems likely. It is reasonable to expect that a primate the size and likely growth rate of *Darwinius* lived a maximum of about 20 years.

### Projected growth remaining to adulthood

Organ systems in *Saimiri* (and by analogy *Darwinius*) mature at different rates: the brain reaches more than 90% of its volume in the first two months, for example, while body weight is added more slowly. Measures of body length are intermediate in growth rate. If we place *Darwinius*, like a *Saimiri*, between the emergence of permanent I_1_ and I_2_, we can expect that she had achieved about 85% of adult head and body length, with growth in foot length slightly ahead of growth in femur length. We have less information about how growth would change the intermembral index, but Young [48] notes that 8–10 month old *Saimiri boliviensis* achieved an intermembral index of 79.9, close to the reported adult value for *Saimiri* of 79.1, so this is unlikely to change substantially.

For a check on these projections, we can compare *Darwinius* to longitudinal growth in *Galago senegalensis*, a more rapidly growing primate. *Galago* mothers carry or park infants for about 7 weeks; weaning is said to be in the range of 70–100 days, with first birth at about a year of age and maximum life span of 16 years [47], [49]. Less is known of tooth emergence, but we can broadly estimate that a juvenile comparable to *Darwinius* in tooth emergence is somewhere near 100 days old [50]. In the captive *Galago* colony studied by Schaefer and Nash [49], an intermembral index of about 60 at birth declined to 57 at 100 days, reaching 55 at full growth. For *Galago*, it appears that relative growth added more to the hindlimb and trunk than to the forelimb. There is however, no universal direction of intermembral index ontogeny; Schaefer and Nash point out that the trend is away from evenness, but that forelimb dominated species like the apes will increase the intermembral index late in life.

Thus, with either *Galago* or *Saimiri* as a living model, we arrive at similar relative place in life history: *Darwinius* was a weaned, independently feeding juvenile with a fraction of growth remaining that might have altered its intermembral index by a percent or two. Further, we would expect that brain and orbit size were near adult values, although some growth remained in face length.

### Locomotion

There are several ways to try to understand locomotion in primates, and these often involve ratios or indices of pairs of measurements. A favorite is the intermembral index (ratio of humerus+radius length divided by femur+tibia length) [51: fig. 10.5], [22: fig. 3.13]. Such indices simplify comparison of proportions to a simple linear scale that is always as dependent on the denominator as it is on the numerator, and simply cannot identify the effects of overall size let alone remove them.

Here we have taken a different approach, compiling measurements of 11 skull, trunk, and limb lengths for 45 species of extant primates, subjecting these to a multivariate principle components analysis (PCA; following [52]). This provides loadings and contrasts that enable functional interpretation of axes, and scores that enable insertion of *Darwinius* masillae to see how it compares. Measurements included are cranial length plus the 10 postcranial measurements listed in Table 2. Species analyzed included Cheirogaleidae (6 species), Lemuridae (9), Lepilemuridae (4), Indriidae (4), Daubentoniidae (1), Galagidae (8), Lorisidae (5), Tarsiidae (1), Callitrichidae (3), and Cebidae (4).

**Figure 13:**
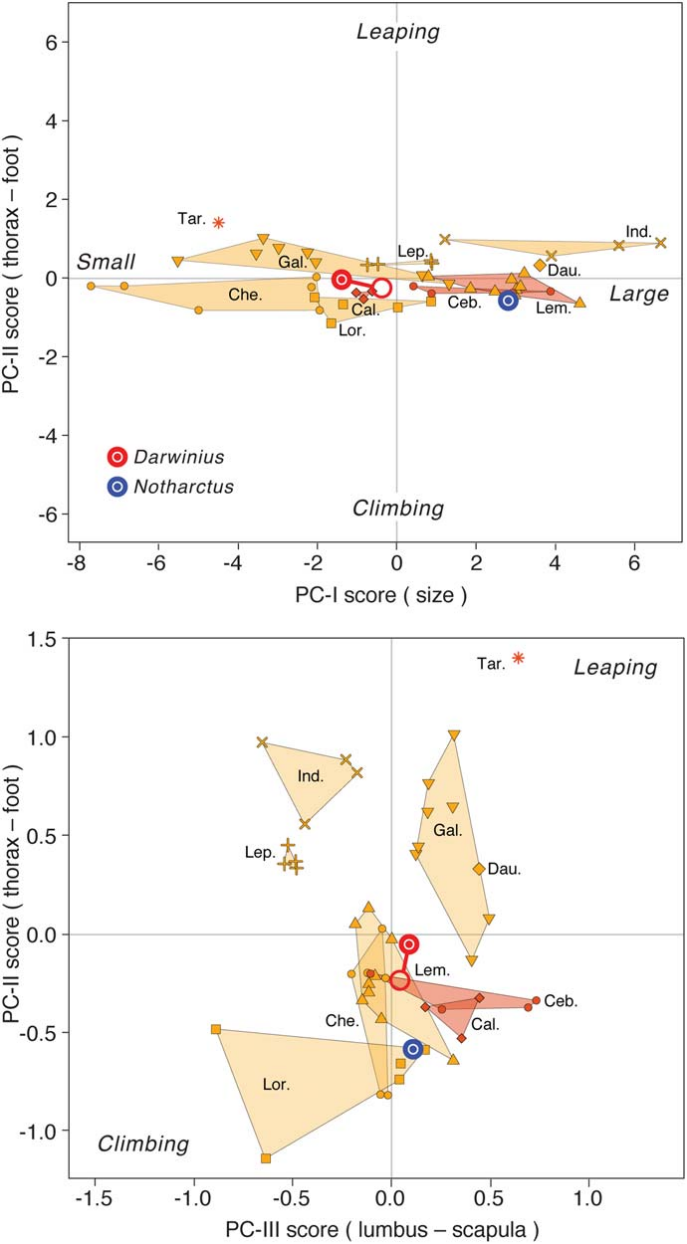
Principle components analysis (PCA) of trunk and limb proportions in extant Lemuroidea, Lorisoidea, Tarsioidea, and Ceboidea. (A)— Bivariate plot of PC-I and PC-II, with both axes drawn to the same scale. All loadings for PC-I are similar and positive, indicating that PC-I represents body size (small primates are at left and larger primates are at right; the coefficient of determination (R^2^) for PC-I and body weight is greater than 0.8). Loadings for PC-II contrast thorax length and foot length, with climbers having a longer thorax and shorter foot, and leapers having a longer foot and shorter thorax. (B)— Bivariate plot of PC-III and PC-II, with both axes drawn to the same scale. Interpretation of PC-II is the same as in A, but here the scale is expanded. Loadings for PC-III contrast lumbus length and scapula length, with climbers having a longer lumbus and shorter scapula, and leapers having a longer scapula and shorter lumbus. *Darwinius* can be projected into this PCA in two ways: as the juvenile it is (filled red circle) or the adult it is projected to become (open red circle; projection computed by augmenting each body segment by the amount it is expected to grow to reach adulthood, using growth curves of [53]. Position of *Notharctus* is shown for comparison, based on measurements in [23]. Family abbreviations: Lemuroidea– *Che*, Cheirogaleiidae; *Dau*, Daubentoniidae; *Ind*., Indriidae; *Lep*, Lepilemuridae. Lorisoidea– *Gal*., Galagidae; *Lor*., Lorisidae. Tarsioidea– *Tar*., Tarsiidae. Ceboidea– *Cal*., Callitrichidae; *Ceb*., Ceboidea. Note that *Darwinius* falls in the middle of both plots, near Callitrichidae in size, and overlapping Lemuridae and Cebidae in trunk and limb proportions. *Darwinius* is interpreted as an arboreal quadruped specialized neither for slow climbing nor for leaping.

*Darwinius* was analyzed both at the size it was when it died (Table 2), and at the size it is expected to have become when it was full grown. The latter required projection using the expected change in proportions of individual body segments. The only source of such information is the compilation by Sirianni and Swindler [53] for *Macaca* (this is not an ideal primate model, but the requisite growth information for primates is rare). Measurements for *Darwinius masillae* are listed in Table 2, and the PCA results are illustrated in Figure 13.

Figure 13A is a bivariate plot of PC-I and PC-II, with both axes drawn to the same scale. All loadings for PC-I are similar and positive, indicating that PC-I represents body size. Loadings for PC-II contrast thorax length and foot length, with climbers having a longer thorax and shorter foot, and leapers having a longer foot and shorter thorax. Figure 13B is a bivariate plot of PC-III and PC-II, again with both axes drawn to the same scale (the latter plot is an enlarged projection of scores looking down the PC-I axis of Fig. 13A). Loadings for PC-III contrast lumbus length and scapula length, with climbers having a longer lumbus and shorter scapula, and leapers having a longer scapula and shorter lumbus. Thus both PC-II and PC-III distinguish leaping from climbing primates.

When *Darwinius* is projected into this PCA, as the juvenile it is (filled red circle) or as the adult it is projected to have become (open red circle), the result is virtually the same. *Darwinius* falls in the middle of both plots, near Callitrichidae in size, and overlapping Lemuridae and Cebidae in trunk and limb proportions. Thus *Darwinius* is interpreted as an arboreal quadruped specialized neither for slow climbing nor for leaping. *Notharctus osbornianus* (filled blue circle) is a larger North American contemporary of *Darwinius masillae*, but it occupies a similarly central position in the PCA.

### Body weight and diet

Body weight is an important parameter of life history and functional morphology [54]. For mammals it is often said that calculations based on cranial and postcranial measurements yield lower and more reasonable body weights than those derived from dental measurements [16: 168], [55], [22: 72]. The advantage with the complete skeleton of *Darwinius masillae* is that it is possible to compare its body size with that of living primates in several different ways. The maximum skull length of *Darwinius masillae*, estimated at 52 mm, would correspond to a body weight of 385 g [50: 383]. Average weight can be predicted from limb segment lengths, and a multiple regression of these [56], yielding 590 and 580 g, respectively, for *Darwinius*. Weights can be predicted by regressing weights for extant primates on their PC-I scores in Figure 13A, and applying the empirically determined relationship to *Darwinius*: This yields an estimated weight of 485 g for *Darwinius* when it died, and a projected body weight of 660 g had it lived to be adult. Molar size (Table 2) gives larger estimates on the order of 1600–1700 g [57]. Thus we have a wide range of body weight estimates for *Darwinius*. It is reasonable to consider that weights based on PC-I scores might be better than measuring the crushed juvenile skull: a weight of 660 g for an adult *Darwinius* is about twice that estimated from skull length alone, and it is on the order of one-half that estimated from tooth size.

Lastly, with a skeleton so complete, we can try something even simpler, matching complete head and body length with a living primate. Based on information in Figure 12, we would project that the present head-body length of about 240 would increase to about 280 mm at adulthood. Adult female primates near such a head-body length include *Lepilemur ruficaudatus* and *Hapalemur griseus*, species for which adult female weight is given as 845 and 892 g, respectively [47].

A body weight of 650–900 g lies above Kay's threshold separating insectivorous primates from those gaining their protein from leaves [58]. Study of the contents of the digestive tract of *Darwinius masillae* recovered from plate B has shown the presence of leaves and a fruit in the digestive tract, while remains of insects are missing [24].

### Orbital size and activity pattern

**Figure 14:**
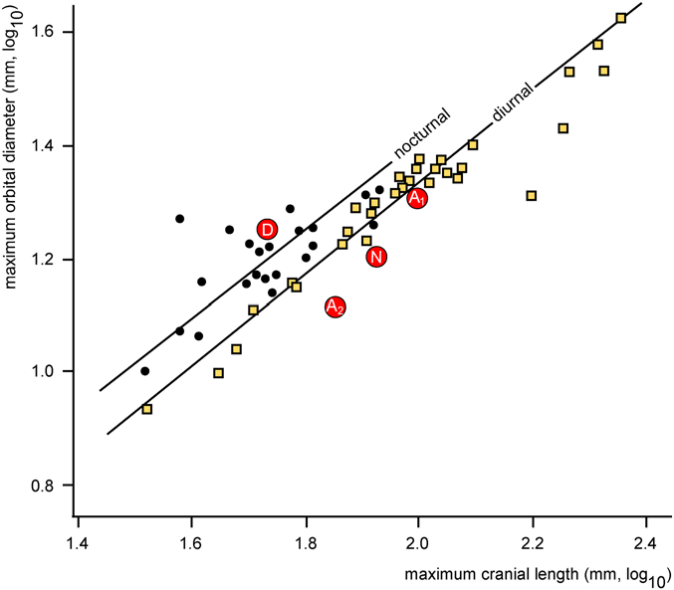
Relative size of the eyes and orbits in *Darwinius* compared to those of other living and fossil primates. *Darwinius* (*D*) has orbits in the nocturnal range (solid circles), while *Adapis magnus* (*A1*), *Adapis parisiensis* (*A2*), and *Notharctus osbornianus* (*N*) are in the diurnal range (open squares). Diagram modified from [1: fig. 10] and [55: 7.6].

The size of the orbit of *Darwinius masillae* can be estimated from both plate A and plate B. These are given in Table 2. The maximal orbital diameter is relatively large compared to skull length, which we interpret as indicating that *Darwinius* was nocturnal [1: 302, fig. 10; Fig. 14]. Remaining facial growth, however, might have slightly reduced relative orbit size had *Darwinius* lived to adulthood.

## Discussion

### Comparative considerations

The overall shape of the *Darwinius* skull is very similar to the Late Eocene North American cercamoniine *Magharita stevensi* as reconstructed by Rasmussen in 1990 [37]. The short rostrum, robust lower jaw, and large braincase look almost the same. The relatively larger orbits of *Darwinius* indicate that the animal could have been nocturnal (Fig. 14). The maxillary suture with the premaxilla and nasal curves in the same way as in *M. stevensi*, displaying a steep premaxilla/maxilla suture and a caudally gently curving maxilla/nasal suture. In *M. stevensi* the ratio of the length from the mesial border of the canine to the mesial border of the orbit divided by the length from the mesial border of the canine to the back of the skull is 1/5 [37]. In the *Darwinius* specimens (plate A and B) the proportions are similar but the flattened specimens are difficult to measure with accuracy. The skull of the European cercamoniine *Pronycticebus gaudryi* is more robust and has a longer braincase and larger orbits (for systematic discussion see [59]). Contrary to large adapid skulls [28], the zygomatic arch is mesially low and slender, and a crista sagittalis was obviously not developed. A well-developed sagittal crest is also present in *Cantius actius* (“*Hesperolemur*” in [59]), but not in *M. stevensi* or in *Europolemur kelleri* [19].

The bony porus acusticus forms a deep channel in *Darwinius*, similar to that known for *M. stevensi* [37] and *Cantius actius* [60]. Although the channel is deep, it is unlike that of anthropoids, lacking a meatus acusticus externus. Its posterolateral portion is formed by the petrosal and not by the ectotympanic. Thus, the condition of the bony ear in *Darwinius* is comparable to that described in the large lemur *Megaladapis edwardsi* [61].

### Phylogenetic Relations

Living primates have long been divided into Strepsirrhini, with a moist nose and median cleft in the upper lip, and Haplorhini, with a dry nose and continuous upper lip. Strepsirrhini was named by Étienne Geoffroy Saint-Hilaire in 1812 [62], who included here six genera: *Indri*, *Lemur*, *Loris*, *Nycticebus*, *Galago*, and *Tarsius*, all sharing ‘sinuous’ noses (*strepsi-rhini*, Gr., bent, twisted noses). Haplorhini (*haplo-rhini*, Gr., simple noses) was named much later by Pocock [63], who separated *Tarsius* from lemurs and lorises and grouped it with higher primates. Pocock classified Lemuroidea (including lorises) and Cheiromyoidea as suborders within Strepsirrhini, and he classified Tarsioidea and ‘Pithecoidea’ (Anthropoidea) as suborders within Haplorhini.

Fossils came into the picture in several ways. Hubrecht [64] featured Cope's Eocene *Anaptomorphus homunculus* as showing that the evolutionary lineage leading to *Tarsius* and apes was of great antiquity. Gregory [65] classified Primates in two suborders, Lemuroidea and Anthropoidea, with the former including Lemuriformes (including the ‘primitive’ Eocene Adapidae), Lorisiformes, and Tarsiiformes (including Eocene ‘Anaptomorphidae’). Finally, Elliott Smith [Smith 1919] emphasized the primitive tarsioid traits retained in the Oligocene anthropoid *Parapithecus* to “establish the truth of the Tarsioid ancestry of the Apes.” The primitive tarsioid traits to which he referred (lower dental formula of 1.1.3.3 and V-shaped mandible) have both proven to be artifacts of breakage [Simons 1972, p. 190].

Fossil tarsioid primates including Eocene Omomyidae and Microchoeridae were elevated to haplorhine status from the beginning for the simple reason that *Tarsius* was included in Haplorhini. Eocene notharctines and adapines have never been considered haplorhines. This is due in part to definitions of Strepsirrhini and Haplorhini that are based on characteristics of the rhinarium that do not preserve in fossils [68], [69], [70], and it is also due to Gregory's [65] inclusion of notharctines and adapines in strepsirrhine Lemuroidea. Any paleontologist who works in early Eocene deposits, however, knows how easy it is to confuse the dentitions of primitive tarsioid and adapoid primates because of their similarity [71], [72], [73].

The complete skeleton of *Darwinius masillae* described here provides an opportunity for a broad comparison to Strepsirrhini and Haplorhini. Table 3 lists 30 anatomical and morphological characteristics commonly used to distinguish extant strepsirrhine and haplorhine primates. These were taken from the standard primate textbook by Fleagle [74], from the classic W. C. Osman Hill monographs on Strepsirrhine and Haplorhini [75], [76], and from additional references listed in Table 3. The distributions of characteristics across the strepsirrhine superfamilies Lemuroidea and Lorisoidea and across the haplorhine superfamilies Tarsioidea, Ceboidea, Cercopithecoidea, and Hominoidea are tabulated by X's (unusually specialized taxa excepted). Standard interpretations of each character as primitive or derived within Strepsirrhini or Haplorhini are listed. Characters that are preserved in *Darwinius masillae* are recorded as present or absent depending on whether they are consistent with the corresponding state in the character list. The final column at the right in Table 3 shows which character states can reasonably be considered synapomorphies of *Darwinius* and either Strepsirrhini or Haplorhini (requiring that states be both derived and present). Some characters may be noted as indeterminate for *Darwinius* because of evidence of convergence, for example, presence of tritubercular molars in extant and early Eocene representatives of Tarsioidea means quadrate molars evolved independently and convergently in Strepsirrhini and most later Haplorhini.

All of the determinate synapomorphies in Table 3 link *Darwinius masillae*, and by implication other Adapoidea, to Haplorhini rather than Strepsirrhini (see also Fig. S7). This is a surprising result, but on reflection the grouping of adapoids like *Notharctus* and *Adapis* with Strepsirrhini [65] was based on retention of primitive characteristics like the free ring-like ectotympanic within the auditory bulla. Consideration of adapoids to be Haplorhini, as tarsioids are, helps to explain why the earliest representatives of both groups are so similar and sometimes confused. Note that *Darwinius masillae*, and adapoids contemporary with early tarsioids, could represent a stem group from which later anthropoid primates evolved, but we are not advocating this here, nor do we consider either *Darwinius* or adapoids to be anthropoids.

As currently conceived, the history of Anthropoidea is traced through the Eocene in somewhat speculatively identified lineages of isolated teeth [e.g., 77], [78]. *Darwinius masillae* shows that it is possible to recover much more complete and informative primate fossils. Most primates in the Eocene, certainly most known from cranial remains, are not anthropoids. Continued recovery of complete skeletal remains, like those of *Darwinius masillae* described here, will help to clarify the systematic position of additional primates relative to the strepsirrhine-haplorhine dichotomy within the order, focus attention on specimens complete enough for phylogenetic interpretation, and define the threshold required for inclusion in Anthropoidea.

### Conclusions

We can now document the history of an extraordinary fossil, here named *Darwinius masillae*. Its two parts, although split by private collectors and dispersed to two continents, are virtually reunited here 26 years after discovery. The fossil, including an entire soft body outline (preserved in the Oslo specimen) as well as contents of the digestive tract (investigated in the Wyoming specimen), documents paleobiology and morphology of an extinct early primate from the Eocene of Germany.

After comparative study, we conclude that the *Darwinius* holotype was a juvenile female, weaned and feeding independently on fruit and leaves in the middle floor of early Middle Eocene rain forest of Messel. She may have been nocturnal. She moved as an agile, nail bearing arboreal quadruped and, although perhaps only 60 percent of adult weight at death (Fig. 12), would have grown to be the size of an adult female *Hapalemur*, in the range of 650–900 g. Her pattern of tooth development shows that her species grew up fairly quickly and suggests that she died before one year of age.

*Darwinius masillae* is now the third primate species from the Messel locality that belongs to the cercamoniine adapiforms, in addition to *Europolemur koenigswaldi* and *E. kelleri*. *Darwinius masillae* is unrelated to *Godinotia neglecta* from Geiseltal, which was much more slenderly built. *Darwinius* and *Godinotia neglecta* are similar, however, in the degree of reduction of their antemolar dentition. Morphological characteristics preserved in *Darwinius masillae* enable a rigorous comparison with the two principal subdivisions of living primates: Strepsirrhini and Haplorhini. Defining characters of *Darwinius* ally it with early haplorhines rather than strepsirrhines. We do not interpret *Darwinius* as anthropoid, but the adapoid primates it represents deserve more careful comparison with higher primates than they have received in the past.

*Darwinius masillae* is important in being exceptionally well preserved and providing a much more complete understanding of the paleobiology of an Eocene primate than was available in the past.

## Supporting Information

Figure S1 Maps showing the provenance of *Darwinius masillae*, new genus and species, from Messel in Germany. Inset map shows the location of the town and fossil locality of Messel near Frankfurt in the southwestern part of Germany. Larger map shows the locations of Messel primates 1–7 (Table 1) within the Messel oil shale excavation. Messel primate 6 near turtle hill is the type of *Darwinius masillae*. It is not known where in the site Messel primate 8, type specimen of *Europolemur kelleri*, was found.
(0.44 MB TIF)

Figure S2 Skull of *Darwinius masillae*, new genus and species. (A)- Detailed photo. (B)- drawing of sutures observed on the skull. (C)- Micro-CT of the skull in plate A, viewed from the right side. Rectangle showing area enlarged in D. (D)-Enlarged view of ear region. Dark grey: petrosal. Abbreviations: bocc-basioccipital, cn- crista nuchualis, fr-frontal, j-jugal, l-lachrymal, M-mandible, mx-maxilla, n-nasale, occ-occipital, p-petrosal, pa- parietal, pmx-premaxilla, sq-squamosal. A-C at same scale.
(9.32 MB TIF)

Figure S3 Skeletal drawing of *Darwinius masillae*, new genus and species, showing the identification of vertebrae. Drawing represents the skeleton visible in plate A (Fig. 1,2). Abbreviations: C, cervical vertebra; T, thoracic vertebra; L, lumbar vertebra; S, sacral vertebra; and Ca, caudal vertebra.
(0.10 MB TIF)

Figure S4 Right forelimb forelimb of *Darwinius masillae*, new genus and species, compared to those of other Eocene primates. (A)- *Notharctus osborni* (after [23]). (B)- *Godinotia neglecta*, holotype (humerus reversed and in cranial view; GMH L-2). (C)-*Europolemur kelleri*, Messel (SMF-ME 1683). (D)- *Europolemur koenigswaldi*, Messel, holotype (SMF-ME 1128). (E)- *Darwinius masillae*, Messel (plate A; PMO 214.214, holotype). Note the relatively short forearm of *Darwinius masillae* compared to those of *Notharctus*, *Godinotia*, and *Europolemur*. The forearm of *Darwinius* is projected to grow only an additional 15%, leaving it well short of the other taxa shown here.
(0.13 MB TIF)

Figure S5 Right hind limb of *Darwinius masillae*, new genus and species, compared to those of other Eocene primates. (A)- *Darwinius masillae*, holotype (plate A; PMO214.214). (B)- *Europolemur kelleri* (HLMD-Me 7430). (C) - *Europolemur koenigswaldi* (SMNK-ME 1125). All are scaled to the same femur length for comparison. The upper and lower leg of *Darwinius* are projected to grow an additional 12%, which would not alter the proportions shown here.
(0.18 MB TIF)

Figure S6 Life restorations of *Darwinius masillae* n. gen., n. sp. Sketches are by Bogdan Bocianowski.
(5.44 MB TIF)

Figure S7 Cladogram to show systematic position of *Darwinius masillae*, n. gen., n. sp. based on characters discussed in the text and numbered in Table 3.
(4.73 MB TIF)

Appendix S1 Measurements of individual bones of *Darwinius masillae*. Tables 4–23.
(0.24 MB DOC)

## References

[1] Franzen JL (2000b). Der sechste Messel-Primate (Mammalia, Primates, Notharctidae, Cercamoniinae).. Senckenbergiana lethaea.

[2] Gingerich PD (1977). Radiation of Eocene Adapidae in Europe.. Géobios, Mémoir special.

[3] Gingerich PD (1979). Phylogeny of middle Eocene Adapidae (Mammalia, Primates) in North America: *Smilodectes* and *Notharctus*.. Journal of Paleontology.

[4] Gingerich PD (1981a). Cranial morphology and adaptations in Eocene Adapidae. 1. Sexual dimorphism in *Adapis magnus* and *Adapis parisiensis*.. American Journal of Physical Anthropology.

[5] Gingerich PD (1975). A new genus of Adapidae (Mammalia, Primates) from the late Eocene of southern France, and its significance for the origin of higher primates.. Contributions of the Museum of Paleontology of the University of Michigan.

[6] Richter G, Storch G (1980). Beiträge zur Ernährungsbiologie eozäner Fledermäuse aus der “Grube Messel”.. Natur und Museum.

[7] Franzen JL, Köster A (1994). Die eozänen Tiere von Messel – ertrunken, erstickt oder vergiftet?. Natur und Museum.

[8] Koenigswald Wv, Braun A, Pfeiffer T (2004). Cyanobacteria and seasonal death: A new taphonomic model for the Eocene Messel lake.. Paläontologische Zeitschrift.

[9] Schaal S, Ziegler W (1992). Messel. An insight into the history of life and of the Earth.

[10] Koenigswald Wv, Koenigswald Wv, Storch G (1998). Ein Halbaffenmännchen..

[11] Gruber G, Micklich N (2007). Messel – Treasures of the Eocene.

[12] Franzen JL (2007). Eozäne Equoidea (Mammalia, Perissodactyla) aus der Grube Messel bei Darmstadt (Deutschland). Funde der Jahre 1969–2000.. Schweizerische Paläontologische Abhandlungen.

[13] Koenigswald Wv (1979). Ein Lemurenrest aus dem eozänen Ölschiefer der Grube Messel bei Darmstadt.. Paläontologische Zeitschrift.

[14] Koenigswald Wv (1985). Der dritte Lemurenrest aus dem mitteleozänen Ölschiefer der Grube Messel bei Darmstadt.. Carolinea.

[15] Franzen JL (1983). Ein neuer Primate aus dem Eozän von Messel. Paläontologische Gesellschaft, 53.. Jahresversammlung, Programm und Kurzfassungen der Vorträge.

[16] Franzen JL (1987). Ein neuer Primate aus dem Mitteleozän der Grube Messel (Deutschland, S-Hessen).. Courier Forschungs-Institut Senckenberg.

[17] Franzen JL (1988). Ein weiterer Primatenfund aus der Grube Messel bei Darmstadt.. Courier Forschungs-Institut Senckenberg.

[18] Franzen JL, Fleagle JF, Kay RF (1994). The Messel primates and anthropoid origins.. Anthropoid origins.

[19] Franzen JL (2000a). *Europolemur kelleri* n.sp. von Messel und ein Nachtrag zu *Europolemur koenigswaldi* (Mammalia, Primates, Notharctidae, Cercamoniinae).. Senckenbergiana lethaea.

[20] Franzen JL (2005). The implications of the numerical dating of the Messel fossil deposit (Eocene, Germany) for mammalian biochronology.. Annales de Paléontologie.

[21] Mertz DF, Renne PR (2005). A numerical age for the Messel fossil deposit (UNESCO World Heritage Site) derived from ^40^Ar/^39^Ar dating on a basaltic rock fragment.. Courier Forschungsinstitut Senckenberg.

[22] Thalmann U (1994). Die Primaten aus dem eozänen Geiseltal bei Halle/Saale (Deutschland).. Courier Forschungsinstitut Senckenberg.

[23] Gregory WK (1920). On the structure and relations of *Notharctus*, an american Eocene Primate. Studies on the Evolution of Primates, Part III.. Memoirs of the American Museum of Natural History (N.S.).

[24] Franzen JL, Wilde V (2003). First gut content of a fossil primate.. Journal of Human Evolution.

[25] Schaller O (2007). Illustrated veterinary anatomy nomenclature.

[26] Hartenberger J-L (1970). Les mammifères d'Egerkingen et l'histoire des faunes de l'Eocène d'Europe.. Bulletin de la Société géologique de France (7).

[27] Rütimeyer L (1862). Eocaene Säugethiere aus dem Gebiet des Schweizerischen Jura.. Neue Denkschriften der allgemeinen Schweizerischen Gesellschaft für die gesammten Naturwissenschaften.

[28] Godinot M, Couette S, Sargis E, Dagosto M (2008). Morphological diversity in the skulls of large adapines (Primates, Adapiformes) and its systematic implications.. Mammalia evolutionary morphology: A tribute to Frederick S. Szalay.

[29] Dean MC, Vesey P (2008). Preliminary observations on increasing root length during the eruptive phase of tooth development in modern humans and great apes.. Journal of Human Evolution.

[30] Hamrick MW, Alexander JP (1996). The hand skeleton of *Notharctus tenebrosus* (Primates, Notharctidae) and its significance for the origin of the primate hand.. American Museum Novitates.

[31] Godinot M, Jouffroy FK, Buffetaut E, Mazin JM, Salmon E (1983). La main d'*Adapis* (Primate, Adapidé).. Actes du symposium paléontologique G. Cuvier, Montbéliard – France 1982.

[32] Godinot M (1992). Early euprimate hands in evolutionary perspective.. Journal of Human Evolution.

[33] Decker RL, Szalay FS, Jenkins FA (1974). The pes in Eocene Adapidae (Lemuriformes, Primates).. Primate locomotion.

[34] Gebo DL (1986). Anthropoid origins – the foot evidence.. Journal of Human Evolution.

[35] Marivaux L, Chaimanee Y, Ducrocq S, Marandat B, Sudre J, Soe AN, Tun ST, Htoon W, Jaeger J-J (2003). The anthropoid status of a primate from the late Middle Eocene Pondaung formation (Central Myanmar): Tarsal evidence.. PNAS.

[36] Dagosto M (1988). Implications of postcranial evidence for the origin of Euprimates.. Journal of Human Evolution.

[37] Rasmussen DT (1990). The phylogenetic position of *Magharita stevensi*. Protoanthropoid or lemuroid?. International Journal of Primatology.

[38] Franzen JL, Frey E (1993). *Europolemur* completed.. Kaupia.

[39] Wuttke M (1983). Weichteil Erhaltung durch lithifizierte Mikroorganismen bei mittel-eozänen Vertebraten aus den Ôlschiefern der Grube Messel bei Darmstadt.. Senckenbergiana Lethaea.

[40] Dixon AF (1987). Baculum length and copulatory behavior in primates.. American Journal of Primatology.

[41] Smith BH, Teaford MFerguson, Smith M (2000). ‘Schultz's Rule’ and the evolution of tooth emergence and replaement in primates and ungulates.. Development, function and evolution of teeth.

[42] Schultz AH, Tanner JM (1971). Age changes in primates and their modification in man.. Human growth III.

[43] Shigehara N (1980). Epiphyseal union, tooth eruption, and sexual maturation in the common tree shrew, with reference to its systematic problem.. Primates.

[44] Smith BH (1991). Age of weaning approximates emergence of the first permanent molar in non-human primates.. American Journal of Physical Anthropology.

[45] Kelley J, Smith TM (2003). Age at first molar emergence in early Miocene *Afropithecus turkanensis* and life-history evolution in the Hominoidea.. Journal of Human Evolution.

[46] Smith BH (1989). Dental development as a measure of life history in primates.. Evolution.

[47] Rowe N (1996). The Pictorial Guide to the Living Primates.

[48] Young JW (2008). Ontogeny of locomotion in *Saimiri boliviensis* and *Callithrix jacchus*: implications for primate locomotor ecology and evolution..

[49] Schaefer MS, Nash LT (2007). Limb growth in captive *Galago senegalensis*: getting in shape to be an adult.. American Journal of Primatology.

[50] Smith BH, Crummett TL, Brandt KL (1994). Ages of eruption of primate teeth: A compendium for aging individuals and comparing life histories.. Yearbook of Physical Anthropology.

[51] Martin RD (1990). Primate origins and evolution. A phylogenetic reconstruction.

[52] Gingerich PD (2003). Land-to-sea transition of early whales: evolution of Eocene Archaeoceti (Cetacea) in relation to skeletal proportions and locomotion of living semiaquatic mammals.. Paleobiology.

[53] Sirianni JE, Swindler DR (1985). Growth and development of the pigtailed macaque.

[54] Soligo C (2006). Correlates of body mass evolution in primates.. American Journal of Physical Anthropology.

[55] Dagosto M, Terranova CJ (1992). Estimating body size of Eocene primates: A comparison of results from dental and postcranial variables.. International Journal of Primatology.

[56] Gingerich PD (1990). Prediction of body mass in mammalian species from long bone lengths and diameters.. Contributions from the Museum of Paleontology, University of Michigan.

[57] Gingerich PD, Smith BH, Rosenberg KR (1982). Allometric scaling in the dentition of primates and prediction of body weight from tooth size in fossils.. American Journal of Physical Anthropology.

[58] Kay RF (1975). The functional adaptations of primate molar teeth.. American Journal of Physical Anthropology.

[59] Godinot M (1998). A summary of adapiform systematics and phylogeny.. Folia primatologica.

[60] Gunnell GF (1995). New notharctine (Primates, Adapiformes) skull from the Uintan (Middle Eocene) of San Diego County, California.. American Journal of Physical Anthropology.

[61] MacPhee RDE (1987). Basicranial morphology and ontogeny of the extinct giant lemur *Megaladapis*.. American Journal of Physical Anthropology.

[62] Geoffroy Saint-HilaireÉ (1812). Suite au tableau des Quadrumanes.. Annales du Muséum d'Histoire Naturelle, Paris.

[63] Pocock RI (1918). On the external characters of the lemurs and of *Tarsius*.. Proceedings of the Zoological Society of London.

[64] Hubrecht AAW (1897). The descent of the primates: Lectures delivered on the occasion of the sesquicentennial celebration of Princeton University.

[65] Gregory WK (1915). I. On the relationship of the Eocene lemur *Notharctus* to the Adapidae and to other primates. II. On the classification and phylogeny of the Lemuroidea.. Bulletin of the Geological Society of America.

[66] Smith GE (1919). Discussion on the zoological position and affinities of *Tarsius*.. Proceedings of the Zoological Society of London.

[67] Simons EL (1972). Primate evolution: an introduction to man's place in nature.

[68] Rosenberger AL, Strasser E, Delson E (1985). Anterior dentition of *Notharctus* and the adapid-anthropoid hypothesis.. Folia Primatologica.

[69] Beard KC (1988). The phylogenetic significance of strepsirhinism in Paleogene primates.. International Journal of Primatology.

[70] Asher RJ (1998). Morphological diversity of anatomical strepsirrhinism and the evolution fo the lemuriform toothcomb.. American Journal of Physical Anthropology.

[71] Simons EL (1962). A new Eocene primate genus, *Cantius*, and a revision of some allied European lemuroids.. Bulletin of the British Museum (Natural History), Geology.

[72] Szalay FS (1976). Systematics of the Omomyidae (Tarsiiformes, Primates): taxonomy, phylogeny, and adaptations.. Bulletin of the American Museum of Natural History.

[73] Gingerich PD (1986). Early Eocene *Cantius torresi*– oldest primate of modern aspect from North America.. Nature.

[74] Fleagle JG (1999). Primate adaptation and evolution, second edition.

[75] Hill WCO (1953). Primates: Comparative anatomy and taxonomy. I– Strepsirhini, a monograph.

[76] Hill WCO (1955). Primates: Comparative anatomy and taxonomy. II– Haplorhini: Tarsioidea, a monograph.

[77] Marivaux L, Antoine P-O, Baqri SRH, Benammi M, Chaimanee Y, Crochet J-Y, de Franceschi D, Iqbal N, Jaeger J-J, Métais G, Roohi G, Welcomme J-L (2005). Anthropoid primates from the Oligocene of Pakistan (Bugti Hills): data on early anthropoid evolution and biogeography.. Proceedings of the National Academy of Sciences USA.

[78] Bajpai S, Kay RF, Williams BA, Das DP, Kapur VV, Tiwari BN (2008). The oldest asian record of Anthropoidea.. Proceedings of the National Academy of Sciences USA.

[79] Long JO, Cooper RW, Rosenblum L, Cooper R (1968). Physical growth and dental eruption in captive-bred squirrel monkeys,*Saimiri sciureus* (Leticia, Columbia).. The squirrel monkey.

[80] Manocha SL (1979). Physical growth and brain development of captive-bred male and female squirrel monkeys, *Saimiri sciureus*.. Experientia.

[81] Pucciarelli HM, Muñe MC, Oyhenart EE, Orden AB, Villanueva ME, Rodriguez RR, Pons ER (2000). Growth of skeletal components in the young squirrel monkey (*Saimiri sciureus boliviensis*): a longitudinal experiment.. American Journal of Physical Anthropology.

[82] Tappen NC, Severson A (1971). Sequence of eruption of permanent teeth and epiphyseal union in New World monkeys.. Folia Primatologica.

[83] Franzen JL (1997). Ein Koprolith als Leckerbissen. Der siebte Primatenfund aus Messel.. Natur und Museum.

[84] Franzen JL, Preuschoft H, Chivers DJ (1993). The oldest primate hands: Additional remarks and observations.. Hands of Primates.

[85] Godinot M, Beard KC (1991). A survey of fossil primate hands and an evolutionary inquiry emphasizing early forms.. Human Evolution.

[86] Godinot M, Beard KC, Preuschoft H, Chivers DJ (1993). A survey of fossil primate hands.. Hands of Primates.

[87] Plavcan JM (2001). Sexual dimorphism in primate evolution.. Yearbook of Physical Anthropology.

[88] Schwartz GT, Samonds KE, Godfrey LR, Jungers WL, Simons EL (2002). Dental microstructure and life history in subfossil Malagasy lemurs.. Proceedings of the National Academy of Sciences USA.

[89] Schmitz J, Ohme M, Zischler H (2001). SINE insertions in cladistic analyses and the phylogenetic affiliations of *Tarsius bancanus* to other primates.. Genetics.

