# Correction: Complete Primate Skeleton from the Middle Eocene of Messel in Germany: Morphology and Paleobiology

**DOI:** 10.1371/annotation/137a79c7-5807-47fc-b885-1f5cc2493305

**Published:** 2009-07-21

**Authors:** Jens L. Franzen, Philip D. Gingerich, Jörg Habersetzer, Jørn H. Hurum, Wighart von Koenigswald, B. Holly Smith

The following subsection should be added beneath the Methods subsection "Terminology":

Nomenclatural Acts

The electronic version of this document does not represent a published work according to the International Code of Zoological Nomenclature (ICZN), and hence the nomenclatural acts contained herein are not available under that Code from the electronic edition. A separate edition of this document was produced by a method that assures numerous identical and durable copies, and those copies were simultaneously obtainable (from May 21st 2009) for the purpose of providing a public and permanent scientific record, in accordance with Article 8.1 of the Code. The separate print-only edition is available on request from PLoS by sending a request to PLoS ONE, 185 Berry Street, Suite 3100, San Francisco, CA 94107, USA along with a check for $10 (to cover printing and postage) payable to "Public Library of Science".

In addition, this published work and the nomenclatural acts it contains have been retrospectively registered in ZooBank (http://www.zoobank.org/) , the proposed online registration system for the ICZN. The ZooBank LSIDs (Life Science Identifiers) can be resolved and the associated information viewed through any standard web browser by appending the LSID to the prefix "http://zoobank.org/". The LSID for this publication is: urn:lsid:zoobank.org:pub:0357A28C-0547-4423-8E5C-A069128DF888

The LSIDs for the Nomenclature Acts are:

Darwinius

urn:lsid:zoobank.org:act:117EE2BF-DCAD-4311-973A-91366AB04003

D. masillae

urn:lsid:zoobank.org:act:C246EC77-E90B-4FB4-B6CB-ADB473A40320

