# Correction: Complete Primate Skeleton from the Middle Eocene of Messel in Germany: Morphology and Paleobiology

**DOI:** 10.1371/annotation/18555b51-1fd1-47b6-a362-acaaa24a53da

**Published:** 2009-07-24

**Authors:** Jens L. Franzen, Philip D. Gingerich, Jörg Habersetzer, Jørn H. Hurum, Wighart von Koenigswald, B. Holly Smith

The authors have supplied an updated Competing Interests statement, which reads as follows:

The authors wish to declare, for the avoidance of any misunderstanding concerning competing interests, that a production company (Atlantic Productions), several television channels (History Channel, BBC1, ZDF, NRK) and a book publisher (Little Brown and co) were involved in discussions regarding this paper in advance of publication. However, to clarify, none of the authors received any financial benefit from any of these associations and these organizations had no influence over the publication of this paper or the science contained within it. The Natural History museum in Oslo will receive some royalty from sales of the book, but no revenue accrues to any of the scientists. In addition, the Natural History Museum of Oslo purchased the fossil that is examined in this paper, however, this purchase in no way influenced the publication of this paper or the science contained within it, and in no way benefited the individual authors. 

