# Supplementary figures and images for: Complete Primate Skeleton from the Middle Eocene of Messel in Germany: Morphology and Paleobiology
Source: PLoS One. 2009 May 19;4(5):e5723. doi: 10.1371/journal.pone.0005723 (PMC2683573; doi:10.1371/journal.pone.0005723)

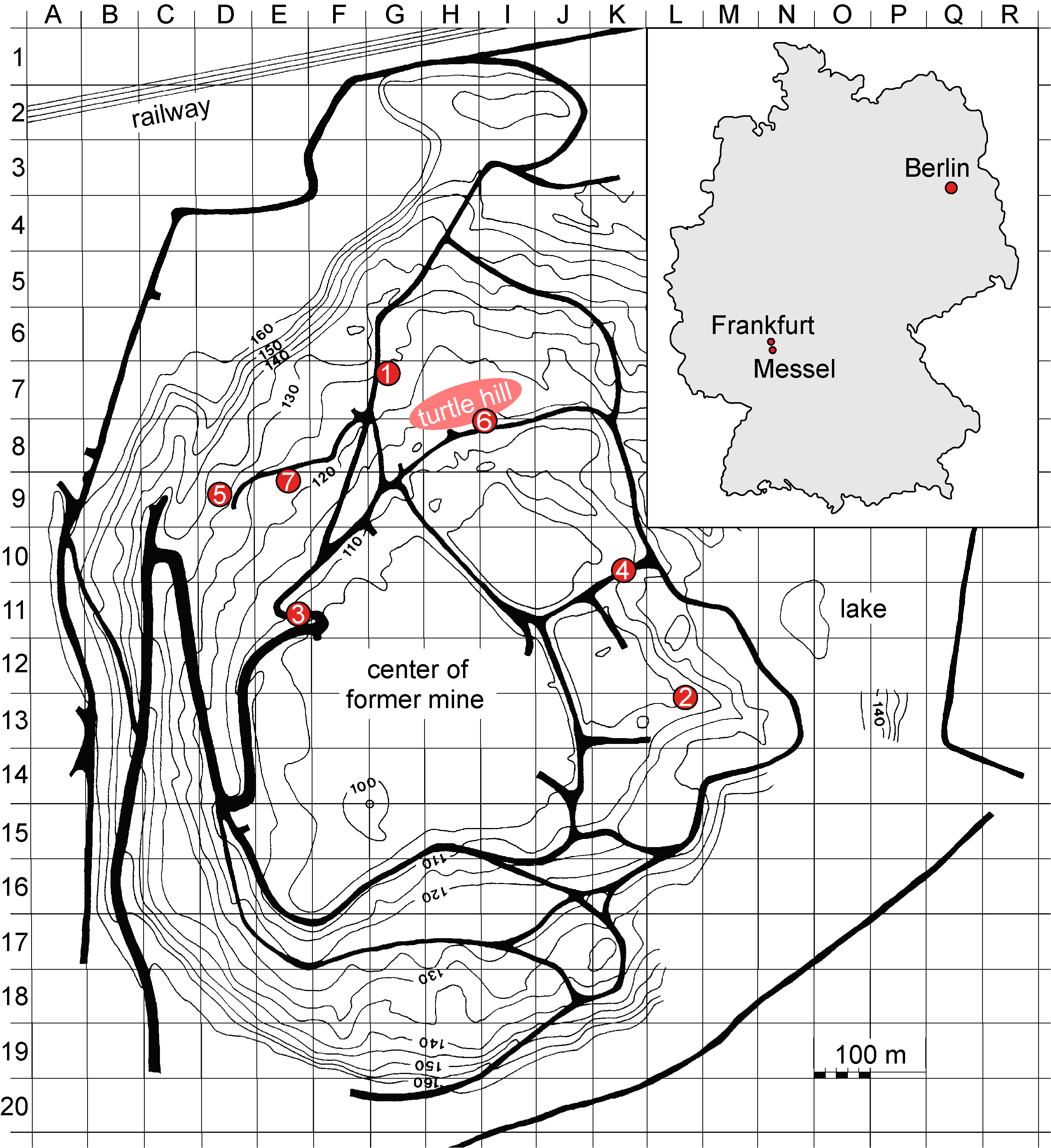

Supplement: Figure S1 — Maps showing the provenance of Darwinius masillae, new genus and species, from Messel in Germany. Inset map shows the location of the town and fossil locality of Messel near Frankfurt in the southwestern part of Germany. Larger map shows the locations of Messel primates 1–7 (Table 1) within the Messel oil shale excavation. Messel primate 6 near turtle hill is the type of Darwinius masillae. It is not known where in the site Messel primate 8, type specimen of Europolemur kelleri, was found. (0.44 MB TIF) [file pone.0005723.s001.tif]

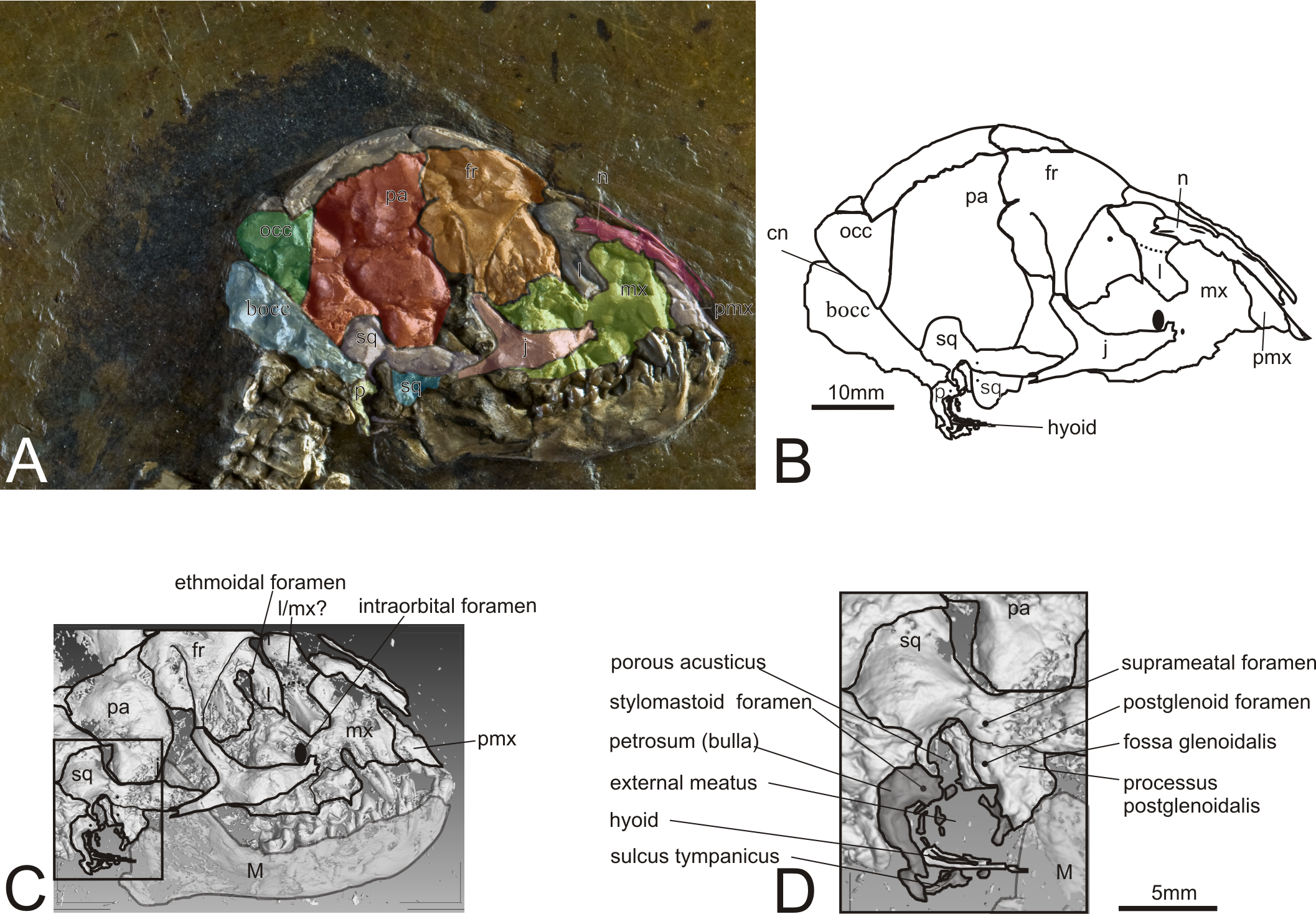

Supplement: Figure S2 — Skull of Darwinius masillae, new genus and species. (A)- Detailed photo. (B)- drawing of sutures observed on the skull. (C)- Micro-CT of the skull in plate A, viewed from the right side. Rectangle showing area enlarged in D. (D)-Enlarged view of ear region. Dark grey: petrosal. Abbreviations: bocc-basioccipital, cn- crista nuchualis, fr-frontal, j-jugal, l-lachrymal, M-mandible, mx-maxilla, n-nasale, occ-occipital, p-petrosal, pa- parietal, pmx-premaxilla, sq-squamosal. A-C at same scale. (9.32 MB TIF) [file pone.0005723.s002.tif]

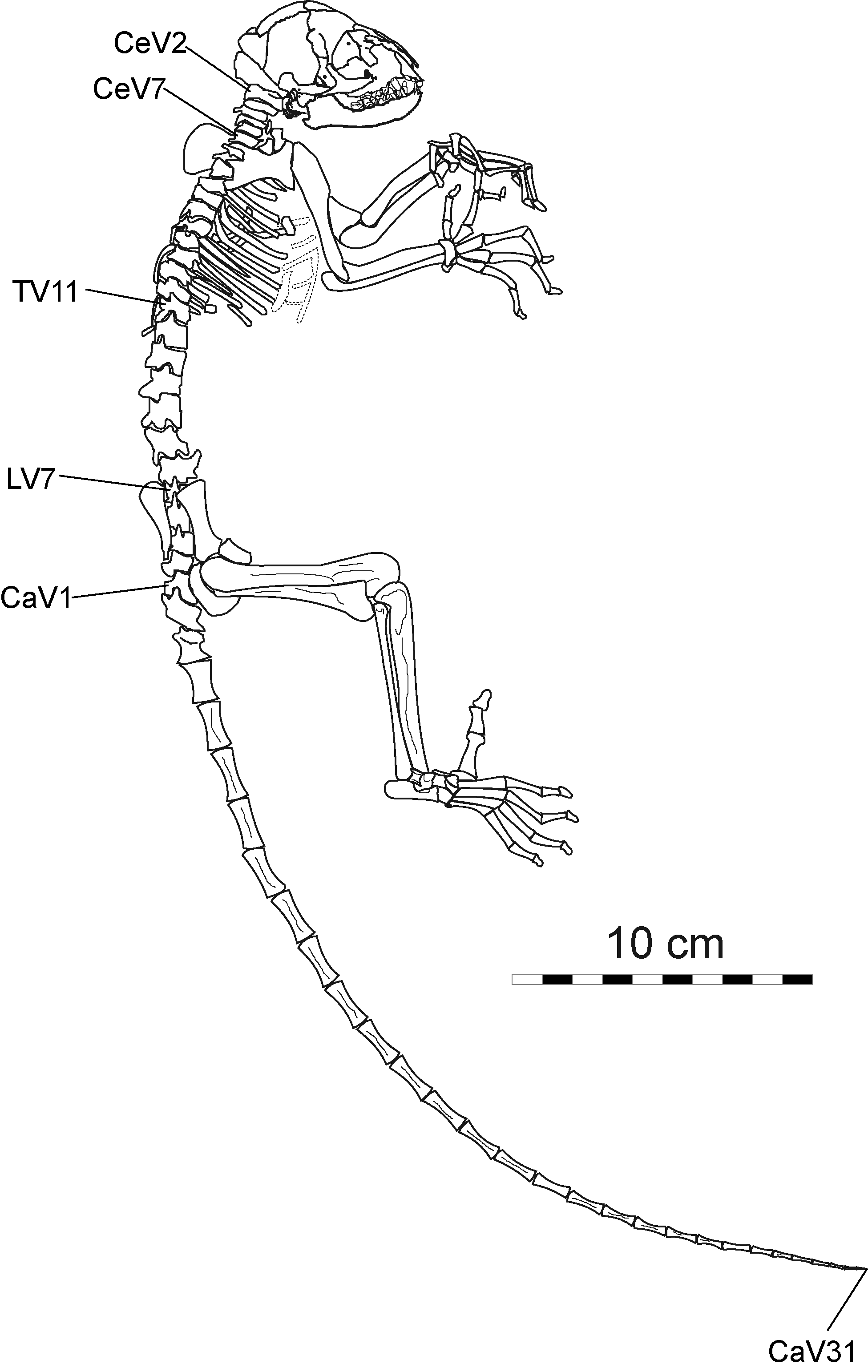

Supplement: Figure S3 — Skeletal drawing of Darwinius masillae, new genus and species, showing the identification of vertebrae. Drawing represents the skeleton visible in plate A (Fig. 1,2). Abbreviations: C, cervical vertebra; T, thoracic vertebra; L, lumbar vertebra; S, sacral vertebra; and Ca, caudal vertebra. (0.10 MB TIF) [file pone.0005723.s003.tif]

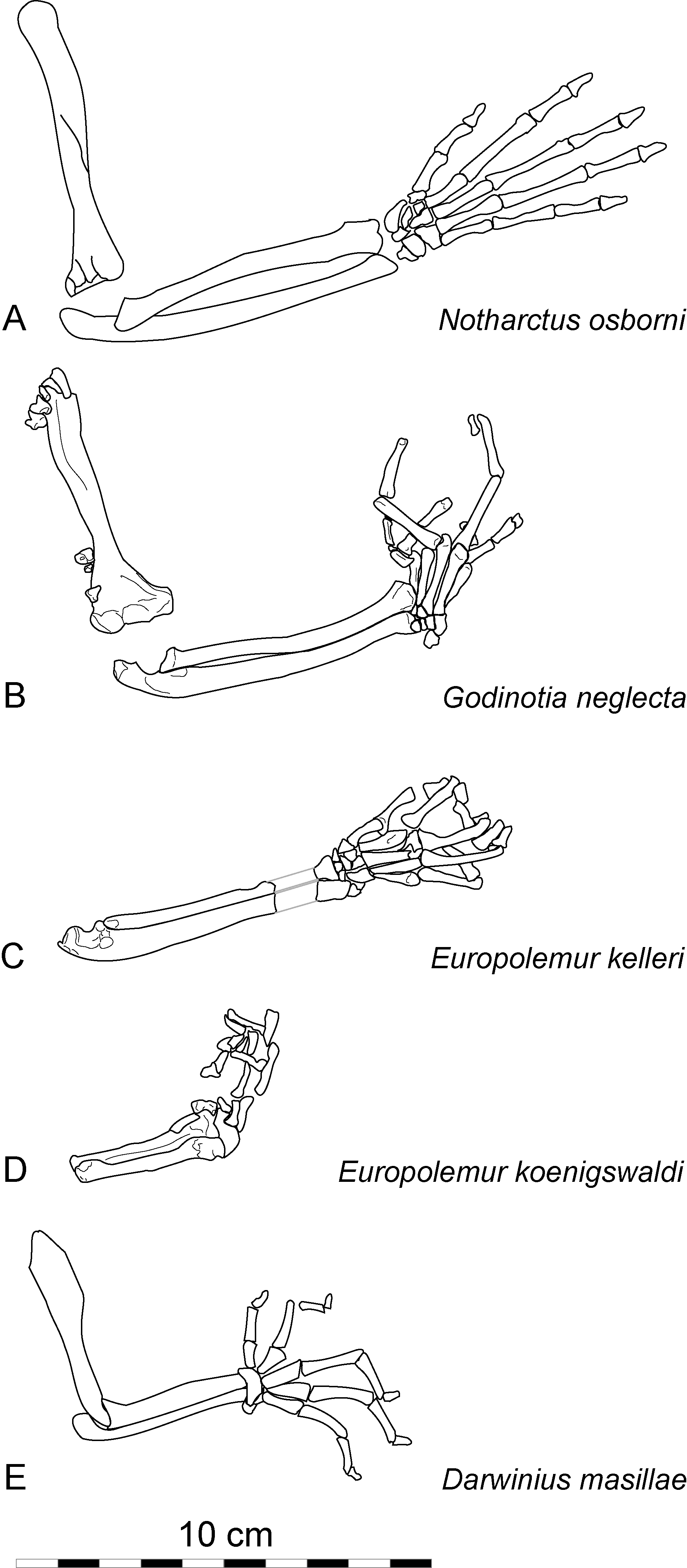

Supplement: Figure S4 — Right forelimb forelimb of Darwinius masillae, new genus and species, compared to those of other Eocene primates. (A)- Notharctus osborni (after [23]). (B)- Godinotia neglecta, holotype (humerus reversed and in cranial view; GMH L-2). (C)-Europolemur kelleri, Messel (SMF-ME 1683). (D)- Europolemur koenigswaldi, Messel, holotype (SMF-ME 1128). (E)- Darwinius masillae, Messel (plate A; PMO 214.214, holotype). Note the relatively short forearm of Darwinius masillae compared to those of Notharctus, Godinotia, and Europolemur. The forearm of Darwinius is projected to grow only an additional 15%, leaving it well short of the other taxa shown here. (0.13 MB TIF) [file pone.0005723.s004.tif]

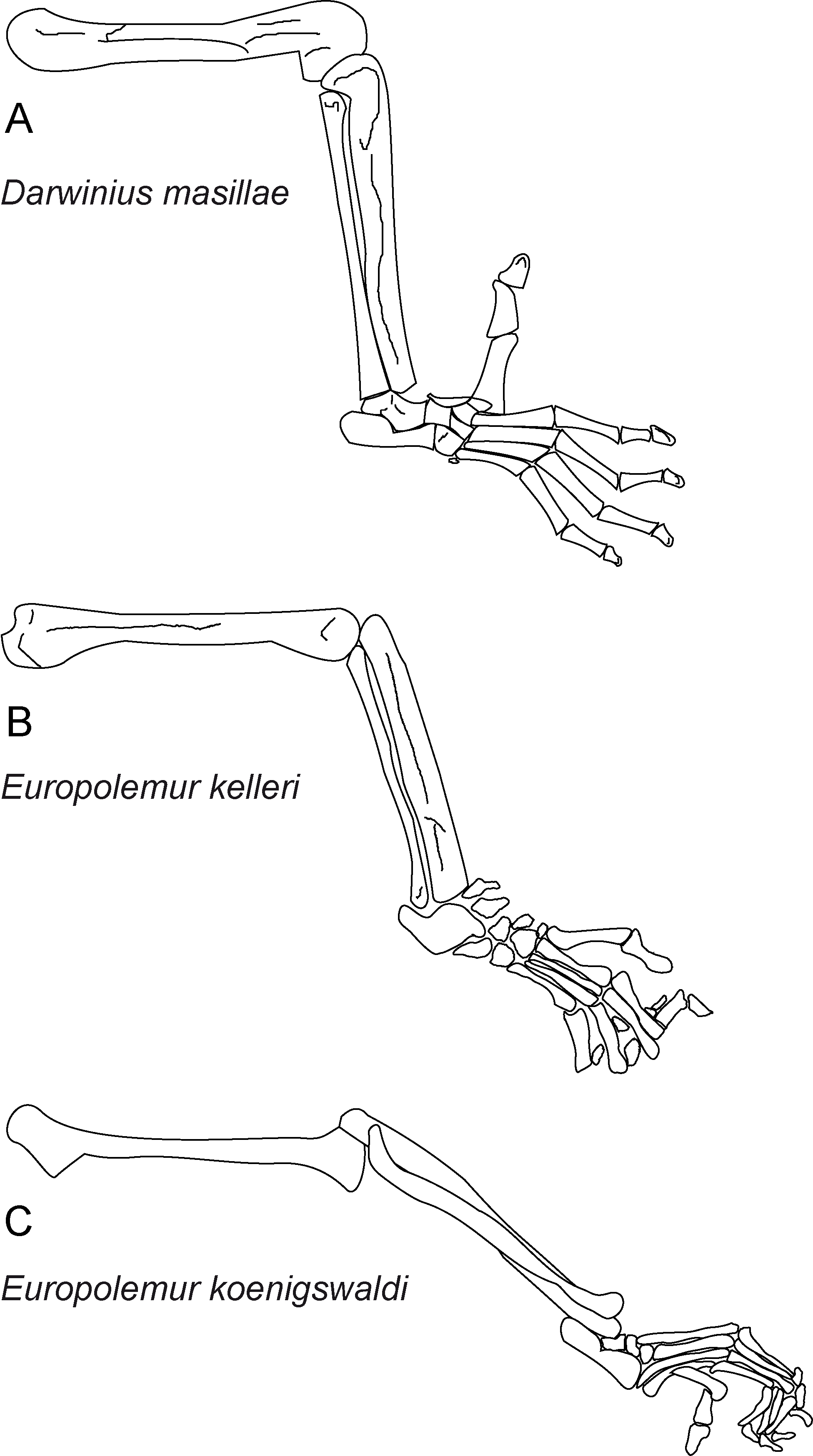

Supplement: Figure S5 — Right hind limb of Darwinius masillae, new genus and species, compared to those of other Eocene primates. (A)- Darwinius masillae, holotype (plate A; PMO214.214). (B)- Europolemur kelleri (HLMD-Me 7430). (C) - Europolemur koenigswaldi (SMNK-ME 1125). All are scaled to the same femur length for comparison. The upper and lower leg of Darwinius are projected to grow an additional 12%, which would not alter the proportions shown here. (0.18 MB TIF) [file pone.0005723.s005.tif]

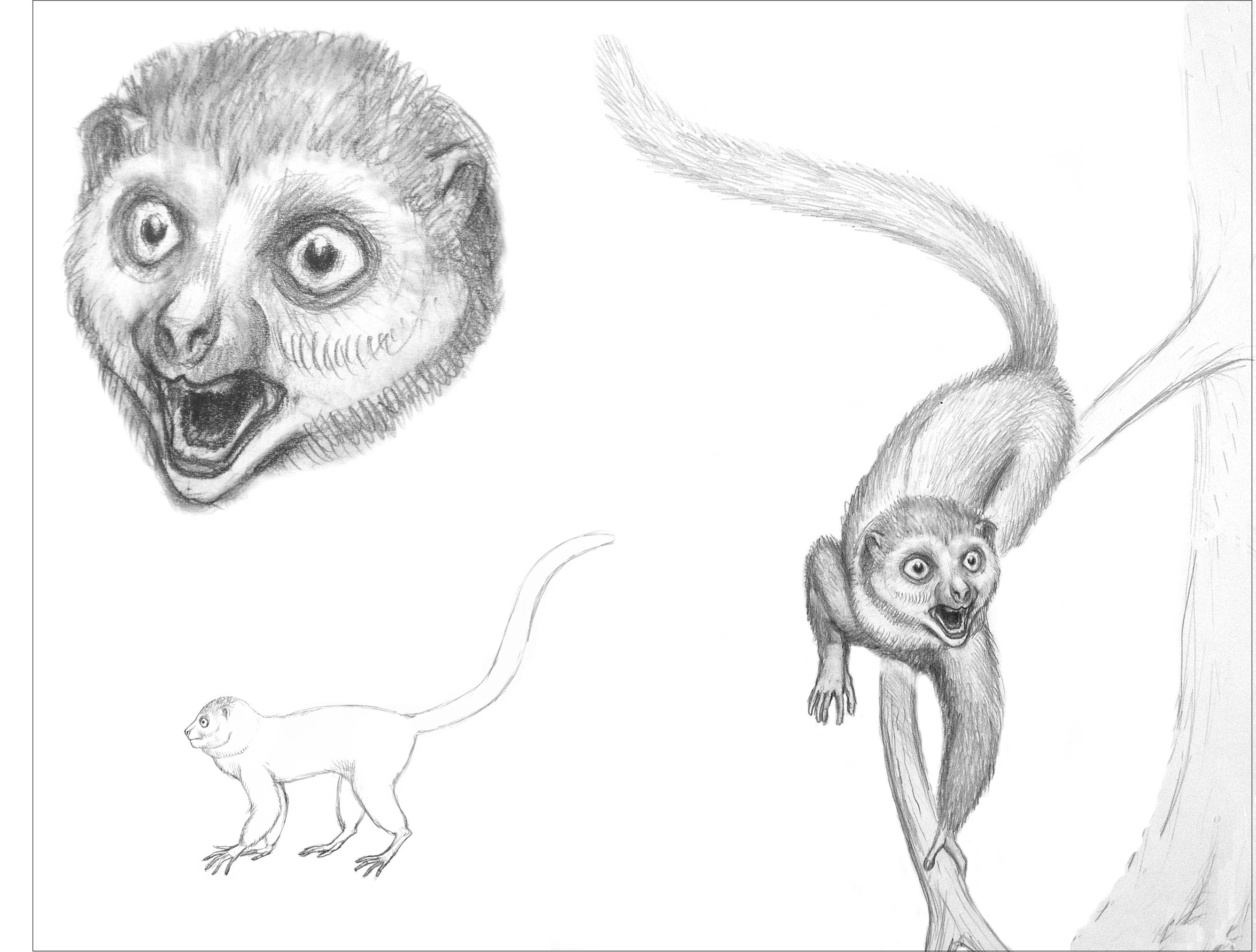

Supplement: Figure S6 — Life restorations of Darwinius masillae n. gen., n. sp. Sketches are by Bogdan Bocianowski. (5.44 MB TIF) [file pone.0005723.s006.tif]

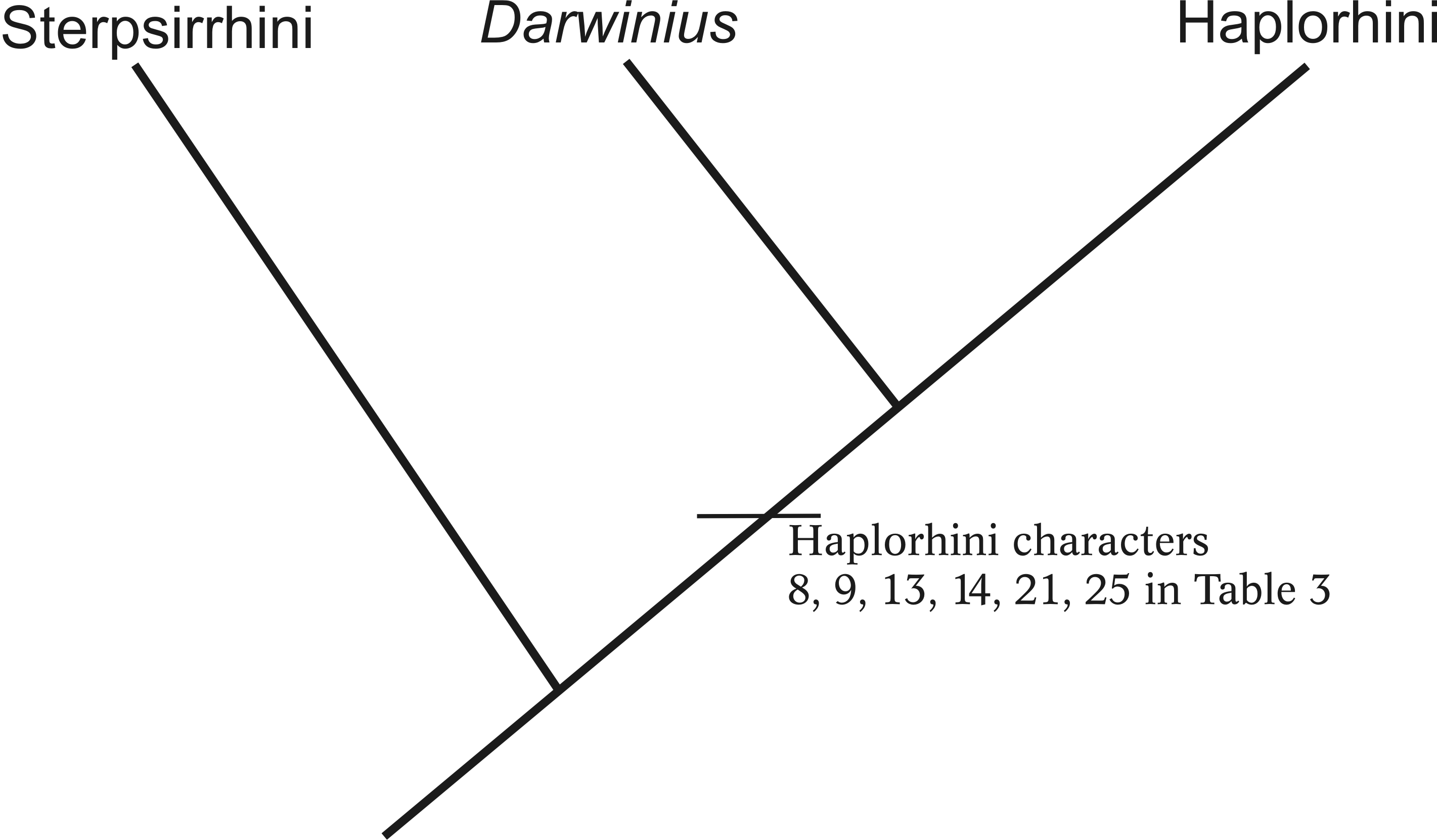

Supplement: Figure S7 — Cladogram to show systematic position of Darwinius masillae, n. gen., n. sp. based on characters discussed in the text and numbered in Table 3. (4.73 MB TIF) [file pone.0005723.s007.tif]
