# Supplementary material for: Complete Primate Skeleton from the Middle Eocene of Messel in Germany: Morphology and Paleobiology
Source: PLoS One. 2009 May 19;4(5):e5723. doi: 10.1371/journal.pone.0005723 (PMC2683573; doi:10.1371/journal.pone.0005723)
Supplement: Appendix S1 — Measurements of individual bones of Darwinius masillae. Tables 4–23. (0.24 MB DOC) [file pone.0005723.s008.doc]

Appendix S1: Tables 4-23

Messel-primates

## Table 4. Skull measurements (mm)

| object /  measurements | *Godinotia neglecta*  (type)  GMH-L-2 | *E. koenigswaldi*  (type)  SMF-ME 1228 | *E. kelleri*  (type)  SMF-ME 3379 | *Darwinius masillae*  plate B  WDC | *D. masillae*  plate A (type)  PMO 214.214 |
| --- | --- | --- | --- | --- | --- |
| maximum length | - | (56.5) | (82) | (52) | (52) |
| basal length | - | - | - | (42) | - |
| preorbital length* | - | (18.5) | (24) | - | 15.1 |
| postorbital length** | - | (25) | (43.5) | - | 27.3 |
| length of nasalia | - | - | - | - | 14.5 |
| free length of nasalia | - | - | - | - | 4.0 |
| depth of nasal incision*** | - | - | - | - | 10.4 |
| orbita: length x height | - | (13) x - | (16.5) x - | (13) x (16.5) | (11.5) x (16.5) |
| mandible, maximum length | - | 44.1 | (57.5) | - | 40.9 |
| mandible, height in front of P_2_ | 7.4 | - | - | 8.4 | 6.8 |
| mandible, height at M_1_/dP_4_ | 10.5 | 7.7 | 9.1/9.3 | 7.2 | 7.3 |
| maximum height of proc. coronoideus | - | - | - | - | 18.2 |
| maximum height of proc. articularis | - | 14.4 | - | - | - |
| height of proc. articularis above occlusal surface | - | (8) | - | (13) | - |

## * from the anterior rim of the orbita to the most mesial point of the premaxilla

## ** from the ventral end of the proc. zygomaticus to the most distal point of the crista nuchalis

## *** from the most distal point of the nasal incision to the most mesial point of the premaxilla

Table 5. Tooth series (mm):

| object /  measurements | *D. masillae*  plate A (type)  PMO 214.214 |
| --- | --- |
| length of dP^4^-P^2^ buccally | 10.4 |
| length of dP_4_-P_2_ | 8.2 |
| length of diastema P^2^-dCsup | 1.0 |
| length of diastema P_2_-dCinf | 0.5 |
| length of diastema dCsup-I^2^ | 2.9 |
| length of diastema dCinf-dI_2_ | 2.5 |

Table 6. Buccal lengths of upper teeth (mm)

| measurements /  object | species | M^3^ | M^2^ | M^1^ | dP^4^ | dP^3^ | P^2^ | dCsup | I^2^ | I^1^ |
| --- | --- | --- | --- | --- | --- | --- | --- | --- | --- | --- |
| Geiseltal, GMH-L-2 | *Godinotia neglecta* s/d | *(3.9)/- | *4.3/- | *(4.0)/- | - | - | *1.3/- | - | - | - |
| SMF-ME 1228 | *E. koenigswaldi* s/d | - | 4.6/4.7 | 4.5 | - | - | - | - | - | - |
| SMF-ME 3379 | *E. kelleri* s/d | 4.6 | 5.6 | 5.9 | 5.3 | 4.5/4.6 | - | - | - | - |
| Pohl | *Darwinius masillae* s | - | - | - | (4.3)/- | 3.4/- | 0.6/- | (2.1)/- | - | - |
| Oslo, PMO 214.214 | *Darwinius masillae* d | - | - | 4.1 | 3.8 | 3.4 | - | 2.3 | 1.5 | 1.5 |

* measured on x-ray

Table 7. Lengths of mandibular teeth (mm)

| tooth position,  measurement/  object | species | M_3_ | M_2_ | M_1_ | dP_4_ | dP_3_ | P_2_ | dCinf | dI_2_ | I_1_ |
| --- | --- | --- | --- | --- | --- | --- | --- | --- | --- | --- |
| Geiseltal, GMH-L-2 | *Godinotia neglecta* s/d | *5.6/*5.8 | *4.8/*4.8 | *4.6/- | - | - | - | -/*2.6 | - | - |
| SMF-ME 1228 | *E. koenigswaldi* s | 6.1 | 5.1 | 4.9 | 4.2 | 3.3 | - | 1.8 | - | - |
| SMF-ME 2986 | *E. koenigswaldi* ? | 5.8 | - | 4.8 | - | - | - | - | - | - |
| SMF-ME 3379 | *E. kelleri* ? | - | 6.2 | 5.1 | 5.1 | 4.0 | - | - | - | - |
| Pohl | *Darwinius masillae* s | - | - | (5.0) | 3.9 | 3.2 | 1.1 | (2.1) | - | - |
| Oslo, PMO 214.214 | *Darwinius masillae* d | - | 4.2 | 4.2 | 3.5 | (3.2) | 0.9 | (1.7) | 0.9 | 1.9 |

* measured on x-ray

## Table 8. Measurements of sacrum (mm)

| measurement/  object | species | L max  median |
| --- | --- | --- |
| SMF-M34718 | *Avahi laniger* | 26.0 |
| SMF-M34719 | *Avahi laniger* | 18.6 |
| Pohl | *Darwinius masillae* | 19.8 |
| Oslo, PMO 214.214 | *Darwinius masillae* | 20.5 |

## Table 9. Measurements of scapula, pelvis and patella (mm)

| bone  measurement/  object | species | scapula | | | | pelvis | | | | | | patella |
| --- | --- | --- | --- | --- | --- | --- | --- | --- | --- | --- | --- | --- |
|  |  | L max | B max | B min  at collum | ∅ max  acetabulum | L max  tuber coxae–  t. ischium | L max  tuber coxae–  t. sacrale | ∅ max  acetabulum | L  preace-  tabular | L  postace-  tabular | B min  ilium | L max |
| SMF-M34718 | *Avahi laniger* | 42.0/42.5 | 18.5/18.0 | 6.8/6.6 | -/6.6 | 63.1/63.3 | 18.8/18.6 | 9.1/9.3 | 43.6/43.4 | 14.3/13.6 | 6.9/6.5 | - |
| SMF-M34719 | *Avahi laniger* | 38.6/- | 15.5/- | 6.2/6.4 | 6.4/- | 55.8/56.0 | 15.2/15.6 | 8.1/8.4 | 38.2/38.1 | 11.8/11.7 | 5.7/- | - |
| Geiseltal, GMH-L-2 | *Godinotia neglecta* s | - | - | - | - | - | - | - | - | - | - | *9.2 |
| SMF-ME 1228 | *E. koenigswaldi* d | 31.0 | 12.6 | - | - | - | - | - | - | - | - | - |
| Oslo s/d, PMO 214.214 | *Darwinius masillae* s/d | - | 17.8/- | -/6.7 | -/9.2 | -/44.9 | - | - | -/25.7 | - | -/5.3 | -/10.5 |

* measured on x-ray

## Table 10. Measurements of anterior stylo- and zeugopodia (mm)

##

| bone  measurement/  object | species | humerus | ulna | | | radius |
| --- | --- | --- | --- | --- | --- | --- |
|  |  | L max | L max | L of olecranon  distal of proc.  anconaeus | L of olecranon-  joint axis | L max |
| NMB-6308 | *Eulemur macaco*, s/d | 82.8/82.6 | 101.9/- | 9.4/- | 11.3/- | 88.8/89.0 |
| NMB-10442 | *Callithrix jacchus*, s/d | 45.1/44.7 | 43.6/43.8 | 4.3/4.4 | 5.0/5.1 | 39.8/40.1 |
| SMF-M34718 | *Avahi laniger* | 63.2/- | 84.5/83.2 | -/5.3 | -/6.9 | -/74.6 |
| SMF-M34719 | *Avahi laniger* | 58.0/59.0 | 78.0/- | 3.4/- | 5.5/- | 68.3/- |
| Geiseltal, GMH-L-2 | *Godinotia neglecta* s/d | *60.2/- | -/*72.1 | *9.2/*8.8 | *11.5/*10.5 | -/*67.6 |
| SMF-ME 1228 | *E. koenigswaldi* d | ca. 45 | - | - | - | ca. 42.5 |
| SMF-ME 1683 d | *E. kelleri* d | - | ca. 67.5 | - | - | ca. 54 |
| Oslo, PMO 214.214 | *Darwinius masillae* s/d | -/(43) | 43.1/ | 6.5/- | 7.5/- | -/38.4 |

## * measured on x-ray

## Table 11. Measurements of endphalange of pollex (mm)

| measurement/  object | species | L max | B max  prox. | B min  diaphysis | B max  trochlea |
| --- | --- | --- | --- | --- | --- |
| Geiseltal, GMH-L-2 | *Godinotia neglecta* s/d | -/*3.8 | -/*3.4 | -/*1.3 | - |
| Oslo s/d, PMO 214.214 | *Darwinius masillae* s/d | 8.1/- | -/3.3 | -/2.1 | -/3.1 |

* measured on x-ray

## Table 12. Measurements of metacarpals I-V (mm)

| bone measurement/  object | mcI | mcII | | | | mcIII | | | | mcIV | | | | mcV | | | |
| --- | --- | --- | --- | --- | --- | --- | --- | --- | --- | --- | --- | --- | --- | --- | --- | --- | --- |
|  | L max | L max | B max  prox. | B min  diaphys. | B max  trochlea | L max | B max  prox. | B min  diaphys. | B max  trochlea | L max | B max  prox. | B min  diaphys. | B max  trochlea | L max | B max  prox. | B min  diaphys. | B max  trochlea |
| *Godinotia neglecta*  Geiseltal, GMH-L-2 s/d | - | *13.9/*14.2 | *3.1/- | *2.5/- | *4.2/- | *(15.0)/- | - | *2.2/*2.1 | -/*4.6 | *12.7/- | *(2.9)/- | *2.0/*2.1 | *3.6/- | *10.3/*10.3 | *2.8/- | *1.8/*2.1 | *2.6/- |
| *E. koenigswaldi*  SMF-ME 1228 d | 7.2 | ca. 11.5 | - | - | - | - | - | - | - | - | - | - | - | - | - | - | - |
| *E. kelleri*  SMF-ME 1683 d | 10.7 | 13.1 | - | 1.8 | - | 15.3 | ca. 4.5 | 2.6 | - | 13.8 | - | 2.3 | - | 13.3 | - | - | - |
| *D. masillae*  Oslo, PMO 214.214 s/d | -/*(10.4) | 13.9/- | -/3.6 | -/2.1 | - | - | -/3.4 | -/2.6 | -/4.9 | - | - | **-** | -(/4.7) | -/10.0 | -/3.3 | -/1.8 | -/(3.9) |

* measured on x-ray

## Table 13. Maximum lengths of posterior stylo- and zeugopodia (mm)

| bone  measurement/  object | species | femur | tibia | fibula |
| --- | --- | --- | --- | --- |
| NMB-6308 | *Eulemur macaco*, s/d | 126.2/125.8 | 118.0/117.4 | 118.0/118.0 |
| NMB-10442 | *Callithrix jacchus*, s/d | 56.0/56.6 | 58.2/57.4 | 53.4/55.4 |
| SMF-M34718 | *Avahi laniger*, s/d | 126.1/127.6 | 113.4/- | 113.1/- |
| SMF-M34719 | *Avahi laniger*, s/d | 117.3/- | 101.1/- | 96.9/- |
| Geiseltal, GMH-L-2 s | *Godinotia neglecta* | - | *80.5 | *(80) |
| SMNK-Me 1125 | *E. koenigswaldi* | (60.9) | (50.6) | - |
| HLD-Me 7430 | *E. kelleri* | (98) | (90) | - |
| SMNK-Me III-1641 | *Europolemur* sp. | (89) | - | - |
| Oslo, PMO 214.214 d | *Darwinius masillae* | -/65.5 | 65.2 | 60.5 |

* measured on x-ray

Table 14. Measurements of tarsal bones (mm)

| bone  measurement/  object | species | calcaneum | | | | | talus | cuboid | navicular | cuneiform-III | cuneiform-II |
| --- | --- | --- | --- | --- | --- | --- | --- | --- | --- | --- | --- |
|  |  | L  max | B  max | H max  tuber | H pr.  coracoides | B min  tuber | L  max | L  max | L  max | L  max | L  max |
| SMF-M34719 | *Avahi laniger*, s | 19.3 | 6.8 | 5.2 | 7.5 | 2.5 | 14.9 | - | - | - | - |
| SMNK-Me 1125 | *E. koenigswaldi* | 15.5 | - | - | - | - | 12.4 | - | - | - | - |
| HLD-Me 7430 | *E. kelleri* | 21.5 | - | - | - | - | - | - | - | - | - |
| Oslo, PMO 214.214 | *Darwinius masillae* | -/18.1 | - | -/6.0 | -/(6.7) | - | -/12.5 | -/7.0 | -/4.4 | -/6.5 | -/4.9 |

## Table 15. Measurements of hallux (mm)

| measurement/  object | species | L max | B max  prox. | B min  diaphysis | B max  trochlea |
| --- | --- | --- | --- | --- | --- |
| Oslo s/d, PMO 214.214 | *Darwinius masillae* | -/16.2 | - | - | - |

## Table 16. Measurements of metatarsals I-V (mm)

| bone  measurement/  object | mtI | mtII | | | | mtIII | | | | mtIV | | | | mtV | | | |
| --- | --- | --- | --- | --- | --- | --- | --- | --- | --- | --- | --- | --- | --- | --- | --- | --- | --- |
|  | L max | L max | B max  prox. | B min  diaphys. | B max  trochlea | L max | B max  prox. | B min  diaphys. | B max  trochlea | L max | B max  prox. | B min  diaphys. | B max  trochlea | L max | B max  prox. | B min  diaphys. | B max  trochlea |
| *E. koenigswaldi*  SMNK-Me 1125 | 12.3/12.3 | 13.9/13.8 | - | - | - | 15.6/15.3 | - | - | - | - | - | - | - | 12.5 | - | - | - |
| *Darwinius masillae*  Oslo, PMO 214.214 s/d | -/*(14.9) | -/16.3 | -/3.1 | -/2.0 | -/3.9 | -/17.1 | -/3.6 | -/2.3 | -/4.1 | 12.3/12.3 | -/(2.5) | -/1.7 | -/(4.4) | -/15.0 | -/2.3 | -/1.6 | -/(3.5) |

* measured on x-ray

Table 17. Measurements of anterior basal phalanges (mm)

| bone  measurement/  object | phI/1 s/d | | | | phII/1 s/d | | | | phIII/1 s/d | | | | phIV/1 s/d | | | | phV/1 s/d | | | |
| --- | --- | --- | --- | --- | --- | --- | --- | --- | --- | --- | --- | --- | --- | --- | --- | --- | --- | --- | --- | --- |
|  | L max | B max  prox. | B min  diaphysis | B max  dist. | L max | B max  prox. | B min  diaphysis | B max  dist. | L max | B max  prox. | B min  diaphysis | B max  distal | L max | B max  prox. | B min  diaphysis | B max  distal | L max | B max  prox. | B min  diaphysis | B max  distal |
| *Darwinius masillae*  Oslo, PMO 214.214 s/d | -/*8.1 | -/*2.7 | -/*1.8 | -/*2.4 | /(11.9) | -/3.5 | 2.0/2.2 | -/2.5 | -/15.6 | -/4.7 | -/2.2 | -/2.4 | /15.4 | - | - | - | -/12.7 | - | - | - |

* measured on x-ray

Table 18. Measurements of posterior basal phalanges (mm)

| bone  measurement/  object | phI/1 s/d | | | | phII/1 s/d | | | | phIII/1 s/d | | | | phIV/1 s/d | | | | phV/1 s/d | | | |
| --- | --- | --- | --- | --- | --- | --- | --- | --- | --- | --- | --- | --- | --- | --- | --- | --- | --- | --- | --- | --- |
|  | L max | B max  prox. | B min  diaphysis | B max  distal | L max | B max  prox. | B min  diaphysis | B max  distal | L max | B max  prox. | B min  diaphysis | B max  distal | L max | B max  prox. | B min  diaphysis | B max  distal | L max | B max  prox. | B min  diaphysis | B max  distal |
| *E. koenigswaldi*  SMNK-Me 1125, s/d | -/11.3 | -/5.5 | -/3.1 | - | -/14.4 | -/3.7 | -/1.7 | -/2.8 | -/15.6 | -/3.9 | -/1.9 | -/2.7 | -/14.9 | -/4.0 | -/2.4 | -/3.3 | /13.6 | -/4.0 | -/2.2 | -/2.8 |
| *Darwinius masillae*  Oslo, PMO 214.214 s/d | /*14.5) | /*(6.0) | -/*(2.6) | /*(4.8) | /*13.5 | -/*3.7 | -/*1.6 | -/*2.3 | /*(15.5) | -/*3.8 | -/*1.7 | - | /*17.3 | -/*3.5 | -/*2.3 | -/*3.7 | /*15.1 | -/*3.6 | -/*2.2 | -/*3.3 |

* measured on x-ray

Table 19. Measurements of anterior intermediate phalanges (mm)

| bone  measurement/  object | phII/2 s/d | | | | phIII/2 s/d | | | | phIV/2 s/d | | | | phV/2 s/d | | | |
| --- | --- | --- | --- | --- | --- | --- | --- | --- | --- | --- | --- | --- | --- | --- | --- | --- |
|  | L max | B max  prox. | B min  diaphysis | B max  distal | L max | B max  prox. | B min  diaphysis | B max  distal | L max | B max  prox. | B min  diaphysis | B max  distal | L max | B max  prox. | B min  diaphysis | B max  dist. |
| Geiseltal, GMH-L-2 | /*2.4 | - | - | - | - | - | - | - | - | - | - | - | - | - | - | - |
| *E. koenigswaldi*  SMNK-Me 1125 | -/8.3 | - | 1.7/- | - | 12.7/12.2 | - | - | - | 12.1/12.2 | - | - |  | - | -/8.5 | - | - |
| *D. masillae*  Oslo, PMO 214.214 s/d | -/*12.3 | -/*(3.3) | -/*1.9 | -/*2.1 | -/*14.8 | -/*4.7 | -/*2.0 | - | -/*15.1 | -/*4.0 | -/*2.1 | -/*3.2 | -/*11.7 | -/*3.2 | -/*1.8 | -/*2.6 |

* measured on x-ray

Table 20. Measurements of posterior intermediate phalanges (mm)

| bone  measurement/  object | phII/2 s/d | | | | phIII/2 s/d | | | | phIV/2 s/d | | | | phV/2 s/d | | | |
| --- | --- | --- | --- | --- | --- | --- | --- | --- | --- | --- | --- | --- | --- | --- | --- | --- |
|  | L max | B max  prox. | B min  diaphysis | B max  distal | L max | B max  prox. | B min  diaphysis | B max  distal | L max | B max  prox. | B min  diaphysis | B max  distal | L max | B max  prox. | B min  diaphysis | B max  distal |
| *E. koenigswaldi*  SMNK-Me 1125 | 8.0/7.9 | - | - | - | -/8.5 | - | - | - | - | - | - | - | (8.5)/- | - | - | - |
| *D. masillae*  Oslo, PMO 214.214 | -/6.7 | -/3.2 | -/1.5 | -/2.4 | -/8.7 | -/3.5 | -/1.7 | -/(2.2) | -/(11.4 | -/3.5 | - | - | -/8.4 | -/3.4 | -/1.6 | -/(2.3) |

Table 21. Measurements of anterior end phalanges (mm)

| bone  measurement/  object | phI/3 s/d | | | | phII/3 s/d | | | | phIII/3 s/d | | | | phIV/3 s/d | | | | phV/3 s/d | | | |
| --- | --- | --- | --- | --- | --- | --- | --- | --- | --- | --- | --- | --- | --- | --- | --- | --- | --- | --- | --- | --- |
|  | L max | B max  prox. | B min  diaphysis | B max  distal | L max | B max  prox. | B min  diaphysis | B max  distal | L max | B max  prox. | B min  diaphysis | B max  distal | L max | B max  prox. | B min  diaphysis | B max  distal | L max | B max  prox. | B min  diaphysis | B max  distal |
| *D. masillae*  Oslo, PMO 214.214 | -/*2.4 | -/2.9 | -/1.3 | -/2.0 | 4.5/4.7 | -/2.3 | -/1.5 | -/1.8 | 4.4/4.8 | -/2.7 | -/1.6 | -/1.9 | -/4.8 | -/2.8 | -/1.7 | -/2.0 | -/3.7 | -/2.2 | -/1.3 | -/1.7 |

* measured on x-ray

Table 22. Measurements of posterior end phalanges (mm)

| bone  measurement/  object | phI/3 s/d | | | | phII/3 s/d | | | | phIII/3 s/d | | | | phIV/3 s/d | | | | phV/3 s/d | | | |
| --- | --- | --- | --- | --- | --- | --- | --- | --- | --- | --- | --- | --- | --- | --- | --- | --- | --- | --- | --- | --- |
|  | L max | B max  prox. | B min  diaphysis | B max  distal | L max | B max  prox. | B min  diaphysis | B max  distal | L max | B max  prox. | B min  diaphysis | B max  distal | L max | B max  prox. | B min  diaphysis | B max  distal | L max | B max  prox. | B min  diaphysis | B max  distal |
| *E. koenigswaldi*  SMNK-Me 1125 | - | - | - | - | 6.3/6.2 | - | - | - | - | - | - | - | - | - | - | - | - | - | - | - |
| HLD-Me 7430  *E. kelleri* | 9.0 | - | - | - | (I9.0) | - | - | - | (6.0) | - | - | - | (6.0) | - | - | - | (6.0) | - | - | - |
| *D. masillae*, Oslo  PMO 214.214 | -/6.8 | -/5.4 | -/2.5 | -/3.1 | -/5.4 | -/2.7 | -/2.4 | -/2.5 | -/4.5 | -/2.7 | -/2.4 | -/2.5 | -/4.5 | -/3.0 | -/1.8 | -/2.4 | -/4.2 | -/2.7 | -/1.6 | -/1.9 |

Table 23. Maximum lengths of caudal vertebrae (mm)

| caudal vertebra no. | 1 | 2 | 3 | 4 | 5 | 6 | 7 | 8 | 9 | 10 | 11 | 12 | 13 | 14 | 15 | 16 | 17 | 18 | 19 | 20 | 21 | 22 | 23 | 24 | 25 | 26 | 27 | 28 | 29 | 30 | 31 |
| --- | --- | --- | --- | --- | --- | --- | --- | --- | --- | --- | --- | --- | --- | --- | --- | --- | --- | --- | --- | --- | --- | --- | --- | --- | --- | --- | --- | --- | --- | --- | --- |
| *A. laniger*  SMF-M34718 | 8.5 | 8.7 | 8.0 | 7.9 | 8.2 | 14.7 | 20.0 | 22.1 | 24.1 | 24.4 | 24.2 | 23.2 | 23.2 | 21.6 | 20.5 | 20.8 | 17.1 | 15.0 | 13.2 | 11.2 | 9.3 | 8.0 | 6.2 | - | - | - | - | - | - | - | - |
| *A. laniger*  SMF-M34719 | 7.4 | 9.0 | 7.7 | 9.0 | 12.7 | 16.0 | 20.0 | 21.3 | 21.5 | 24.0 | 21.6 | 20.6 | 20.3 | 20.5 | 18.0 | 17.5 | 15.7 | 13.9 | 11.9 | 9.0 | 6.8 | 5.3 | 4.8 | 3.1 | - | - | - | - | - | - | - |
| ***Notharctus osborni* | 8.4 | 8.2 | 8.5 | 10.4 | - | - | 17.4 | - | - | - | - | 25.5 | - | 24.6 | 25.5 | - | - | - | - | - | - | - | - | - | - | - | - | - | - | - | - |
| *E. koenigswaldi*  SMNK-Me 1125 | 3.4 | 4.2 | 7.1 | 9.2 | 9.4 | 10.9 | 11.0 | 11.2 | 12.5 | 11.7 | 11.7 | 11.6 | 10.7 | 10.2 | 10.2 | 9.7 | 8.8 | 8.5 | 7.2 | 6.8 | 5.9 | 5.7 | 4.9 | 4.6 | 4.1 | 3.3 | 2.9 | 2.2 | 1.8 | 0.9 | - |
| *D. masillae*, Oslo  PMO 214.214 | 10.4 | 10.4 | 10.3 | 9.7 | 13.9 | 16.1 | 17.9 | 17.9 | 17.9 | 17.9 | 16.9 | 16.0 | 16.0 | 15.6 | 15.5 | 14.4 | 14.3 | 12.0 | 11.4 | 10.6 | 10.1 | 8.8 | 8.9 | 8.1 | 7.7 | 7.1 | 6.5 | 5.7 | 5.1 | 4.2 | 3.8 |

* measured on x-ray

** measures from Gregory 1920
